# MicroRNA-145 Regulates Human Corneal Epithelial Differentiation

**DOI:** 10.1371/journal.pone.0021249

**Published:** 2011-06-20

**Authors:** Sharon Ka-Wai Lee, Yufei Teng, Hoi-Kin Wong, Tsz-Kin Ng, Li Huang, Peng Lei, Kwong-Wai Choy, Yingpeng Liu, Mingzhi Zhang, Dennis Shun-Chiu Lam, Gary Hin-Fai Yam, Chi-Pui Pang

**Affiliations:** 1 Department of Ophthalmology and Visual Sciences, The Chinese University of Hong Kong, Hong Kong, China; 2 Department of Obstetrics and Gynaecology, The Chinese University of Hong Kong, Hong Kong, China; 3 Joint Shantou International Eye Centre, Shantou, China; University of Pittsburgh, United States of America

## Abstract

**Background:**

Epigenetic factors, such as microRNAs, are important regulators in the self-renewal and differentiation of stem cells and progenies. Here we investigated the microRNAs expressed in human limbal-peripheral corneal (LPC) epithelia containing corneal epithelial progenitor cells (CEPCs) and early transit amplifying cells, and their role in corneal epithelium.

**Methodology/Principal Findings:**

Human LPC epithelia was extracted for small RNAs or dissociated for CEPC culture. By Agilent Human microRNA Microarray V2 platform and GeneSpring GX11.0 analysis, we found differential expression of 18 microRNAs against central corneal (CC) epithelia, which were devoid of CEPCs. Among them, miR-184 was up-regulated in CC epithelia, similar to reported finding. Cluster miR-143/145 was expressed strongly in LPC but weakly in CC epithelia (*P* = 0.0004, Mann-Whitney U-test). This was validated by quantitative polymerase chain reaction (qPCR). Locked nucleic acid-based *in situ* hybridization on corneal rim cryosections showed miR-143/145 presence localized to the parabasal cells of limbal epithelium but negligible in basal and superficial epithelia. With holoclone forming ability, CEPCs transfected with lentiviral plasmid containing mature miR-145 sequence gave rise to defective epithelium in organotypic culture and had increased cytokeratin-3/12 and connexin-43 expressions and decreased ABCG2 and p63 compared with cells transfected with scrambled sequences. Global gene expression was analyzed using Agilent Whole Human Genome Oligo Microarray and GeneSpring GX11.0. With a 5-fold difference compared to cells with scrambled sequences, miR-145 up-regulated 324 genes (containing genes for immune response) and down-regulated 277 genes (containing genes for epithelial development and stem cell maintenance). As validated by qPCR and luciferase reporter assay, our results showed miR-145 suppressed integrin β8 (ITGB8) expression in both human corneal epithelial cells and primary CEPCs.

**Conclusion/Significance:**

We found expression of miR-143/145 cluster in human corneal epithelium. Our results also showed that miR-145 regulated the corneal epithelium formation and maintenance of epithelial integrity, via ITGB8 targeting.

## Introduction

In adult tissue, the renewal of epithelium relies on the population of stem cells. They generate transit-amplifying (TA) cells, which proliferate and differentiate to stratified squamous epithelium. Adult stem cells are usually slow-cycling *in vivo* whereas TA cells are frequently dividing with short cell cycles. When placed in culture, stem cells and TA cells generate holoclones and paraclones respectively [1]. Corneal epithelial progenitor cells (CEPCs) reside in the basal epithelium of limbus which is an annulus located at the vascularized junction between transparent cornea and opaque sclera [2]. They are characterized by a lack of cytokeratin-3/12 and connexin-43, which are corneal differentiation markers [3], [4]. They undergo more frequent cell divisions than differentiated epithelial cells and can be cultured *ex vivo* from limbal tissues [5]. There has been persistent success in clinical application of limbal grafting or autologous limbal culture cells to restore damaged corneal epithelia [6], [7].

Epigenetic factors, such as microRNAs, are known to affect stem cell biology, including the maintenance of pluritotency and differentiation [8], [9]. MicroRNAs are small non-coding RNAs of 20 to 25 nucleotides in length and usually act as endogenous repressor of gene activity [10]. They bind to the 3′ untranslated region (3′UTR) of target mRNAs for translational repression or mRNA cleavage. More than 10,000 distinct microRNA sequences from genomes of viruses, worms and mammals have been identified through random cloning and sequencing or computational prediction (microRNA Registry, http://www.microrna.sanger.ac.uk/sequences). In human, more than 800 microRNAs, attributing to about 2% of known protein coding genes, are known to regulate various biological processes, although many of the target genes remain to be identified.

In mouse, miR-134 induces ES cells to differentiate towards ectodermal lineage [11]. The miR-17-92 cluster maintains the undifferentiated property of lung epithelial progenitor cells [12]. P63, a proliferation regulator of epithelial cells is a target gene of miR-203 [13]. In mammalian eyes, six retina-specific microRNAs (miR-96, 182, 183, 184, 210 and 140-AS) have been identified by microarray analysis [14]. In human and rat retinas, eleven microRNAs (miR-7, 7d, 23a, 29, 107, 124, 135a, 135b, 143, 200b and 206) were identified by a target finding approach on the 3′UTR of known retinal genes [15]. In mouse cornea, miR-184 is highly enriched in basal corneal epithelium but absent in the superficial cells of cornea, whole limbal and conjunctival epithelia [16]. In contrast, miR-205 and 217 are present in corneal, limbal and conjunctival epithelia, and epidermis. MiR-184 may participate in the terminal differentiation of corneal epithelia and antagonize with miR-205, which down-regulates SH2-containing inositol phosphatase-2 in regulating epithelial cell proliferation [17]. In this study, we investigated the microRNA expression in 2 anatomical distinct human corneal tissues: limbal-peripheral corneal (LPC) epithelium containing CEPCs and central corneal (CC) epithelium without CEPCs.

## Methods

### Corneal specimen collection and CEPC culture

Human corneal rims from adult donors were recruited in the Joint Shantou International Eye Centre (JSIEC), China. The JSIEC Independent Ethics Committee approved the study and participants gave written informed consent. The specimens were immediately processed for cryosectioning. LPC and CC epithelia, separated by 1-mm in width, were dissected for small RNA extraction (Fig. S1A). For CEPC isolation, the limbal epithelium was digested with Dispase (50 mg/ml, Invitrogen, Carlsbad, CA, US) and D-sorbitol (100 mM) in SHEM [DMEM/Ham's F-12 (Invitrogen) containing 5 µg/ml transferrin, 5 µg/ml insulin, 5 ng/ml selenate, 0.1 mM ethanolamine, 0.1 mM o-phosphoethanolamine, 5 µg/ml hydrocortisone, 0.5% dimethylsulfoxide, 10 ng/ml recombinant human basic fibroblast growth factor (bFGF, Invitrogen), 10 ng/ml human epidermal growth factor (EGF, Invitrogen), penicillin G and streptomycin sulfate (Invitrogen)]. The epithelium was disintegrated to single cells using TryPLE (Invitrogen) [18] for plating out at 500 cells/cm^2^ on Primaria dish (BD, Franklin Lakes, NJ, US) cultured in CnT20 medium and supplements (CELLnTEC, Basel, Switzerland). Holoclones formed in 7 days were harvested for CEPCs (Fig. S1B). Unless specified, all reagents were obtained from Sigma (St Louis, MI, US).

### Microarray experiments

Total RNA was extracted by Trizol/chloroform and purified with the RNeasy kit (Qiagen, Valencia, CA, US). RNA quantity and integrity were examined by RNA 6000 Pico Chip Kit (Agilent, Santa Clara, CA, US) on Agilent 2100 Bioanalyzer. RNA samples with 28S/18S ratios in the range of 1.4 to 1.8 were used. MicroRNA profiling was performed using Agilent Human microRNA Microarray V2 platform, which screens for the expression of 723 human microRNAs from Sanger database v.10.1. For gene expression analysis, Agilent Whole Human Genome Oligo Microarray was used. RNA samples were labeled with cyanine-3 (Cy3) using the Agilent One–Color Labeling kit and hybridized to the array according to the manufacturer's protocol. The signal was detected with an Agilent DNA Microarray Scanner. The array data is MIAME compliant and the raw data was deposited in NCBI Gene Expression Omnibus (GSE24979 and GSE24980, http://www.ncbi.nlm.nih.gov/geo/).

### Data normalization and analysis

The scanned images were extracted with Feature Extraction Software 9.5.3.1 (Agilent). Background intensity and feature non-uniform outliers were removed by standard procedures. The processed data were imported to GeneSpring GX10.0.2 for log_2_ transformation. Signal cut-off measurements were less than 0.01, and normalized to 75^th^ percentile of signal intensity to standardize each chip for cross-array comparison. Differentially expressed miRNAs were identified using unpaired Student's t test with P values cut off by 0.01 and fold change more than 2.0. MicroRNA target gene prediction was performed by TargetScan algorithm. The gene expression list was uploaded to web-based tool DAVID (Database for Annotation, Visualization and Integrated Discovery) V6.7 for enriched Gene Ontology terms and significant pathway analysis. The statistically significant terms calculated by Fishers Exact T test associated with the biological processes were selected for comparison.

### Real-time quantitative PCR

Complementary DNA (cDNA) was prepared using High Capacity cDNA Reverse Transcription kit (Applied Biosystems, Calsbad, CA, US) from total RNA (1 µg) using Taqman miRNA-specific RT primer (Exiqon, Vedbaek, Denmark, Table S1) and a cocktail containing reverse transcription buffer, dNTP mix, recombinant RNase inhibitor, Multiscribe reverse transcriptase at standard procedure. Real-time PCR was performed using Taqman Universal PCR Master Mix on PRISM 7900HT Sequence Detection System (Applied Biosystems). Amplification of a single fragment was confirmed by a dissociation curve with good correlation with standards and threshold-cycle values. Gene expression was performed by Sybr Green assay or semi-quantitative RT-PCR and primers were listed in Table S2.

### Locked nucleic acid (LNA)-based in situ hybridization

Cryosections (10 µm thick) were fixed in 4% paraformaldehyde, acetylated with acetic anhydride/triethanolamine (Sigma) and treated with 5 µg/ml proteinase K [19]. After pre-hybridization, the sections were hybridized with denatured digoxigenin (DIG)-labeled LNA-miRCURY oligo probe (Exiqon) for specific microRNAs or scrambled sequences at 60°C for 24 hours. After washings, signals were detected by anti-DIG-alkaline phosphatase conjugate (Roche, Basel, Switzerland) followed by reduction with substrate (nitroblue tetrazolium/5-bromo-4-chloro-3-indolyl phosphate, NBT/BCIP, Roche). The sections were counterstained with hematoxylin before examination under light microscopy (DMRB, Leica, Vertrieb, Germany).

### Cell transfection

Human CEPCs from passage 1 holoclones were collected and plated for transfection with lenti-miR plasmid (System Biosciences, MountainView, CA, US) driven by CMV promoter using Lipofectamine 2000 (Invitrogen). Insert sequence for pMIRH143PA-1: 5′-GCGCAGCGC CCUGUUCCCAGCCUGAGGUGCAGUGCUGCAUCUCUGGUCAGUUGGGAGUCUGAGAUGAAGCACUGUAUAGCUCAGGAAGAGAGAAGUUGUUCUGCAG; for pMIRH145PA-1: 5′-CACCUUGUCCCUCACGGGGUCCAGUUUUCCCAGGAAUCCCUUAGAUGCUAAGA UGGGGAUUCCUGGAAAUACUGUCUUGAGGUCAUGGUU. The control was pCDH-CMV-MCS-EF1-copGFP expression vector (System Biosciences). The transfection efficiency was monitored by the percentage of green fluorescent protein (GFP) positive cells.

### Immunofluorescence and western blotting

For immunofluorescence, the samples were fixed with 2% neutral buffered paraformaldehyde and permeabilized with 0.15% saponin (Sigma). After blocking, they were incubated with monoclonal antibody recognizing cytokeratin-3/12 (AE5, Sigma), connexin-43 (Cnx43, Millipore, Billerica, MA, US) or ITGB8 (Sigma), followed by fluorescein-conjugated IgG secondary antibody (Invitrogen) and DAPI (4′,6-diamidino-2-phenylindole) staining. For western blotting, the cells were lysed in 50 mM Tris-HCl containing 150 mM sodium chloride, 1% Nonidet P-40, 0.25% sodium deoxycholate, protease inhibitor cocktail (Roche) and 1 mM phenylmethyl sulfonylfluoride for 30 minutes on ice. The clear supernatant was collected for protein denaturation in a buffer at a final concentration of 2% sodium dodecylsulfate (SDS), 50 mM DL-dithiothreitol and 1% glycerol and analyzed by 10% SDS-PAGE (polyacrylamide gel electrophoresis) using monoclonal antibody against Cnx-43, ABCG2 (Abcam), p63α (Cell Signaling, Danvers, MA, US) or GAPDH (Sigma), followed by appropriate horseradish peroxidase-conjugated Ig secondary antibodies. Staining signals were detected by enhanced chemiluminescence (ECL, GE Healthcare). Except stated, all reagents were obtained from Sigma.

### Corneal epithelium organotypic culture

Human amniotic membrane (AM) obtained by elective Caesarean with written consent was preserved sterile in 50% glycerol at −80°C. Prior to culture, AM epithelium was removed by treatment with 5 mg/ml dispase. The remaining basement membrane and stroma were placed in a culture insert (Corning, Corning, NY, US) with the epithelial side facing up. CEPCs transfected with pre-miRs or scrambled sequences were seeded at a density of 5×10^4^ cells/cm^2^ in serum-free SHEM supplemented with EGF and bFGF and cultured until confluence. The cell monolayer was air-lifted with basal side nourished by culture medium for 21 days to induce multilayer formation. The constructed epithelium was fixed with 10% neutral buffered formaldehyde, paraffin embedded and sectioned for histological examination. Sections were used for immunohistochemical staining with anti-human ITGB8 antibody and horseradish peroxidase-DAB (3,3′-diaminobenzidine) reaction.

### Luciferase reporter assay

A 5734 base-pair XhoI/Not1 fragment encompassing the full-length 3′UTR of the human *ITGB8* gene was amplified by PCR, forward primer: 5′-GAAGCTCGAGCTTTCGGTGCAACT TCTAAA, and reverse primer: 5′-ATTAGCGGCCGCGATTAACACCTACTACTAAACAG. The fragment was ligated into the XhoI/Not1 sites of psiCHECK™-2 vector (Promega, Madison, WI, US) with *Renilla* luciferase as the primary reporter gene. The wildtype pCHECK-ITGB8_3′UTR construct was used as template to generate specific substitution (AACT to TTCT) of miR-145 target site using QuikChange II Site-Directed Mutagenesis kit (Stratagene, La Jolla, CA, US) and oligonucleotides (28–34^th^ site: GATTTTTAAACACTTAATGGGATTCT GGAATTGTTAATAATTGC; and 4421–4427^th^ site: TCTCACTTTTAAACAAAATTTTCT GGAAAAATATTACATGG). Wildtype and mutant constructs were verified by direct sequencing. For the luciferase assay, HeLa cells in 24-well plates were transfected with 0.5 µg construct and 50 pmol of pre-miR-145 or scrambled sequences using Lipofectamine 2000. At 24 hours post-transfection, cells were collected for measuring the luciferase activity by the Dual Luciferase Reporter Assay (Promega). The experiment was repeated 5 times. Mean activities and standard deviation were calculated and samples compared for statistical significance using paired Student's t-test.

## Results

### Demarcation of human limbal-peripheral versus central corneal epithelia

To assess the quality of corneal specimens, one-eighth of each cornea rim with intact limbus was paraformaldehyde-fixed. Cryosections were obtained for immunofluorescence of corneal progenitor and differentiation markers, including p63α, ATP binding cassette glycoprotein member 2 (ABCG2), cytokeratin-15 (CK15), cytokeratin-3/12 (CK3/12), Cnx43 and epidermal growth factor receptor (EGFR) [20]–[23]. Those specimens exhibiting correct expression and localization of these markers were used for further experiments (Figure S2). The cell membrane staining of ABCG2 was observed in limbal basal epithelial cells but not in any of the peripheral and central corneal epithelial cells. The nuclear p63α staining was strong in limbal basal and suprabasal cells, gradually restricting to the basal epithelia of peripheral and central cornea. Undifferentiated CK15 was prominent in limbal basal, limbal suprabasal and peripheral corneal basal epithelia but absent in central corneal basal epithelium. Corneal differentiation marker CK3/12 was absent in limbal basal epithelium and weak in the peripheral and central corneal basal epithelia. Gap junction protein Cnx43 was not expressed in most basal cells of limbus but was positive in central cornea. Cell membranous EGFR was found in basal cells of both limbus and cornea. The exclusive existence of p63α^strong^ABCG2^+^CK3/12^−^Cnx43^−^ cells in basal LPC epithelia demonstrated the existence of CEPCs. Holoclone formation was obtained from cells dissociated from LPC but not from CC, substantiating the presence of CEPCs in LPC (Fig. S1B).

### MicroRNA profiling in human corneal epithelium

From 10 ìm thick cryosections, LPC and CC epithelia were dissected out as pairs and processed immediately for small RNA extraction to preserve the *in situ* microRNA expression, which could be disrupted due to *in vitro* manipulation. Both LPC and CC samples were analyzed for ABCG2 and p63α expression by qPCR and western blotting. The sample pairs with positive ABCG2 expression in LPC but negative in CC were selected for microRNA analysis (Figs. S1C, D).

By qPCR analysis, we found similar expressions of reported housekeeping microRNAs (U6, hsa-let-7a, miR-16 and miR-26b) in both LPC and CC samples (hsa-miR-16 was shown in Fig. S3). The expression of reported ES cell-specific microRNAs (hsa-miR-302a, 302d, 320, 338, 371, 372, 373 and 373#) was also examined. After normalization with housekeeping U6, they were negligibly expressed in both LPC and CC samples. In addition to miR-205 reported to express constitutively in mouse corneal, limbal and conjunctival basal epithelia [16], we also observed similar expressions of ocular-specific hsa-miR-182 and 204 in LPC and CC samples (Fig. S3).

**Figure 1:**
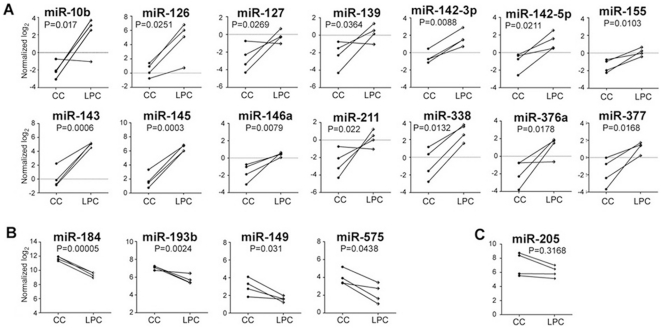
MicroRNA microarray analysis of human LPC and CC epithelial samples. (A) MicroRNAs expressed with >2 folds higher in LPC compared to CC epithelia. (B) MicroRNAs expressed higher by >2 folds in CC compared to LPC epithelia. (C) miR-205 expression in both epithelia. Data were log(2) transformed and normalized to 75^th^ percentile of signal intensity for standardization. Statistical significance was calculated by unpaired Student's *t* test.

Since CEPCs are present in LPC epithelia but not in CC and they could be regulated by microRNAs, we predicted that LPC epithelia might contain microRNAs distinguishable from those in CC epithelia. For microRNA profiling using microarray investigation, we studied 4 pairs of LPC and CC samples showing similar miR-205 expression levels (Fig. 1C). We identified 14 microRNAs (miR-10b, 126, 127, 139, 142-3p, 142-5p, 143, 145, 146a, 155, 211, 338, 376a and 377) expressed by more than 2 folds in LPC than in CC epithelia (*P*<0.05, unpaired Student's *t* test) (Table 1, Fig. 1A). Among them, miR-145 (43.6 folds, *P* = 0.00029) and 143 (27.2 folds, *P* = 0.0006) were the most significantly up-regulated microRNAs in LPC epithelia. On the other hand, 4 microRNAs (miR-149, 184, 193b and 575) were expressed 2 folds or more in CC than in LPC epithelia (*P*<0.05, unpaired Student's *t* test) (Table 1, Fig. 1B). Among them, miR-184, as previously reported [16], was the most significantly up-regulated microRNA in CC epithelia (4.9 folds, *P* = 0.00005, unpaired Student's *t* test).

**Table 1:** List of candidate miRNAs identified from miRNA microarray analysis.

	MicroRNAs	*Unpaired Student's t-test*	Reported functions (incl.)
***Candidate microRNAs up-regulated in LPC versus CC epithelia***			
1	hsa-miR-145	*P* = 0.00029	Tumor suppressor [41], ES pluripotency [25], cardiac SMC differentiation [27]
2	hsa-miR-143	*P* = 0.0006	Tumor suppressor [41], ES pluripotency [25], cardiac SMC differentiation [27]
3	hsa-miR-146a	*P* = 0.0078	Tumor suppressor [42], inflammation [43]
4	hsa-miR-142-3p	*P* = 0.0088	Immune reaction [44], hematopoiesis [45]
5	hsa-miR-155	*P* = 0.0102	Cell metabolism [46], immune reaction [47], viral infection [48]
6	has-miR-10b	*P* = 0.0127	Tumorigenesis and metastasis [49]
7	hsa-miR-338	*P* = 0.0132	Tumor suppressor [50], neurogenesis [51]
8	hsa-miR-377	*P* = 0.0168	Extracellular matrix modelling [52]
9	hsa-miR-376a	*P* = 0.0178	No report
10	hsa-miR-142-5p	*P* = 0.021	Hematopoiesis [53]
11	hsa-miR-211	*P* = 0.022	Tumorigenesis [54]
12	hsa-miR-126	*P* = 0.025	Angiogenesis, cardiac development [55]
13	hsa-miR-127	*P* = 0.0269	Tumorigenesis [56], apoptosis, organ development [57]
14	hsa-miR-139	*P* = 0.0364	Tumorigenesis [58], FoxO1 signaling [59]
***Candidate microRNAs down-regulated in LPC versus CC epithelia***			
1	hsa-miR-184	*P* = 0.00005	Oncogenic [60], neural stem cell-specific [61], cornea-specific [16]
2	hsa-miR-193b	*P* = 0.0024	Tumor suppressor [62]
3	hsa-miR-149	*P* = 0.031	No report
4	hsa-miR-575	*P* = 0.043	No report

**Figure 2:**
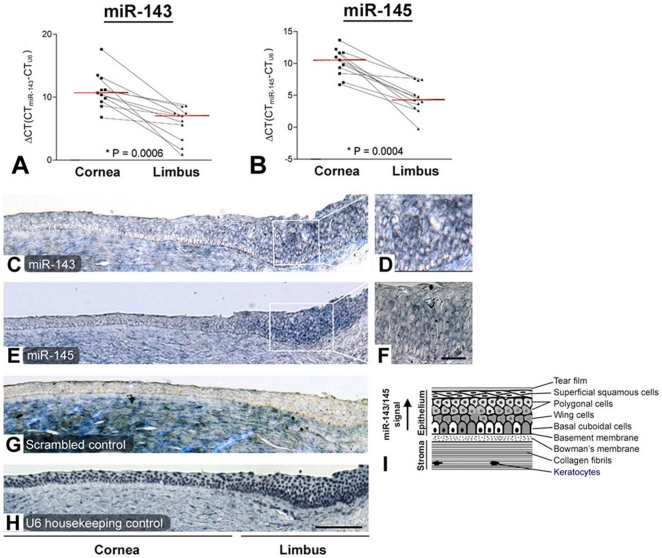
Validation of miR-143 and miR-145 expression in limbal epithelium. By qPCR analysis, (A) miR-143 and (B) miR-145 was up-regulated (compared to U6 expression) in LPC epithelia, when compared to CC (*P*<0.001, Mann Whitney U-test). Red lines indicated mean CT value. *In situ* hybridization showed (C) miR-143 and (E) miR-145 in limbal epithelium, compared to (G) scrambled sequences and (H) U6. At higher magnification, (D) miR-143 and (F) miR-145 were present in parabasal layers. Scale bars: 150 µm (C, E, G, H); 40 µm (D, F).

Elevated expression of miR-143 and miR-145 in LPC epithelia was validated by qPCR on additional 11 pairs of human LPC and CC epithelia. After normalization with the respective U6, ΔCT of miR-143 was 5.9±0.8 in LPC and 11.1±0.9 in CC epithelia (*P* = 0.0006, Mann Whitney U-test) (Fig. 2A). Similarly, ΔCT of miR-145 was 4.5±0.7 in LPC and 10.2±0.7 in CC epithelia (*P* = 0.0004, Mann Whitney U-test) (Fig. 2B). The smaller the ΔCT values relative to U6, the higher the expression. With about 88% efficiency in our PCR amplification system, LPC had miR-143 and miR-145 expressions 26.6-fold and 36.5-fold higher than CC epithelia respectively. Such levels were comparable to the array results described earlier.

Localization of miR-143 and miR-145 in human corneal rim specimens was shown by LNA-based *in situ* hybridization. We hybridized the corneal rim cryosections with denatured DIG-labeled LNA-miRCURY oligo probes for miR-143 (Fig. 2C), miR-145 (Fig. 2E), scrambled sequences (Fig. 2G) and U6 (Fig. 2H). In contrast to U6 as the positive control and scrambled sequence as the negative control, miR-143 and miR-145 were more intensively detected in the limbal epithelium, but low to negligible in the corneal epithelium. Similar results were found in 4 repeated experiments. As shown in Figures 2D and F under higher magnification, miR-143 and miR-145 were present predominantly in the parabasal layers, with the intensity reducing towards the superficial layers. They were not strongly expressed in the basal layer, which contains CEPCs.

### MiR-145 regulated corneal epithelial differentiation

**Figure 3:**
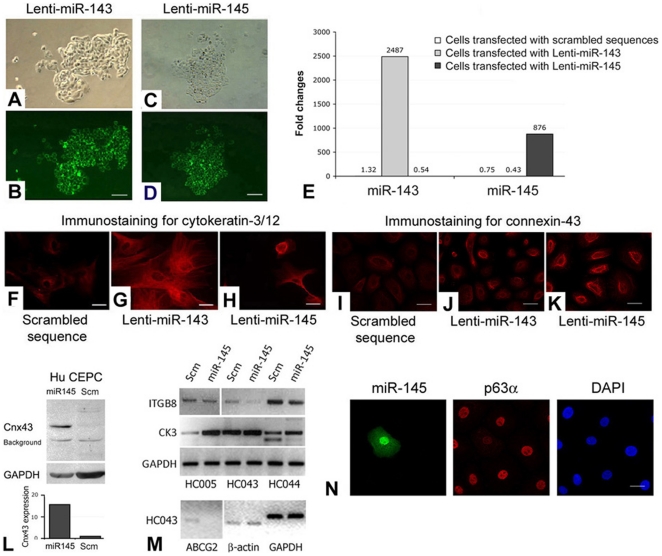
Transfection analysis of miR-143 and miR-145. (A–D) Human P2 CEPCs transfected with (A and B) Lenti-miR-143 and (C and D) Lenti-miR-145. (A, C) Phase-contrast images; (B, D) live GFP imaging. (E) Overexpression levels of miR-143 and 145 in transfected CEPCs by qPCR analysis. Amplification signals from cells with scrambled sequences (Scm) were indicated. Immunofluorescence of (F–H) cytokeratin-3/12 and (I–K) connexin-43 in CEPCs transfected with (F, I) scrambled sequence, (G, J) Lenti-miR-143 and (H, K). (L) Western blotting and densitometry analysis of connexin-43 (Cnx43) and GAPDH in CEPCs transfected with Lenti-miR-145 or scrambled sequences. (M) RT-PCR result of integrin β8 (ITGB8), cytokeratin-3 (CK3), ABCG2, β-actin and GAPDH in different primary CEPCs (at P2) transfected with Lenti-miR-145 or scrambled sequences. (N) Immunofluorescence of miR-145 (revealed by GFP), p63α and nuclear DAPI stain in P2 CEPCs after Lenti-miR-145 transfection. Scale bars: (A–D) 100 µm; (F–K, N) 10 µm.

**Figure 4:**
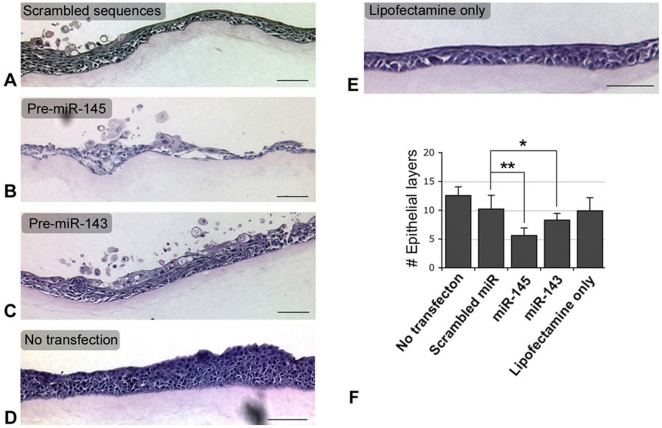
Corneal epithelial organotypic assay. Representative hematoxylin-eosin stained pictures from serial sections of cell-denuded AM composite showing the thickest epithelium and the most epithelial layers. (A) CEPCs transfected with scrambled sequences, (B) CEPCs transfected with pre-miR-145, (C) CEPCs transfected with pre-miR-143 and (D) CEPCs with Lipofectamine 2000 and (E) non-transfected CEPCs. Scale bars: 100 µm. (F) Epithelium forming efficiency determined by the number of epithelial layers in 15 sites along the composite. * *P* = 0.042; ** *P* = 0.0002 (paired Student's t-test).

Freshly isolated human CEPCs were cultured in CnT20 medium and cells from primary holoclones were pooled and plated as passage 2 (P2) for transfection with lenti-miR expression plasmid pMIRH143PA-1 or pMIRH145PA-1 at a ratio of 3 µl Lipofectamine 2000 per µg DNA. Over-expressions of miR-143 and miR-145 were shown by GFP live imaging (Figs. 3A–B, miR-143; 3C–D, miR-145) and qPCR (Fig. 3E). The transfected cells were kept in CnT50 medium with low bovine pituitary extract (15 µg/ml) for optimal corneal epithelial cell growth for 2 days followed by expression analysis. By immunofluorescence, both miR-143 and 145-transfected cells had increased CK3/12 expression (Figs. 3G and H), when compared to cells transfected with scrambled sequences (Fig. 3F). This was also detected by western blotting (Fig. 3N, second panel). On the other hand, miR-145-transfected cells showed relatively stronger Cnx-43 expression (Fig. 3K), which was mild in cells transfected with scrambled sequences (Fig. 3I) or miR-143 (Fig. 3J). Western blot analysis showed Cnx-43 upregulation in CEPCs transfected with miR-145 by about 15 folds more than those with scrambled sequences (Fig. 3L). In addition, these cells had reduced ABCG2 (Fig. 3M, bottom panel) and p63α expression (Fig. 3N) as revealed by RT-PCR and immunofluorescence, respectively. This expression pattern of corneal differentiation markers, i.e., reduced ABCG2 and p63α expression, and its expression in parabasal layers of limbal epithelium, indicated that miR-145 might be involved in corneal epithelial differentiation. We corroborated this supposition by a three-dimensional corneal epithelial organotypic assay. Human P2 CEPCs transfected with miR-143, miR-145 or scrambled sequences were expanded to monolayer cell sheet on denuded AM in submerged culture, followed by air-lifting to induce cell stratification. The composites were harvested for morphological examination. The number of epithelial layer was quantified in 15 random sites along the composite to obtain the epithelium forming efficiency. CEPCs without transfection (Fig. 4D), with lipofectamine only (Fig. 4E) or transfected with scrambled sequences (Fig. 4A), generated thicker epithelia. They had typical epithelial morphology with basal cuboidal-like cells next to the basement membrane. The cells were packed and appeared squamous in shape at the superficial layers (non-transfected: 12.5±1.5 layers; lipofectamine-only; 9.9±2.3 layers; transfected with scrambled sequence: 10.2±2.4 layers) (Fig. 4F). However, this was not observed in the epithelia generated from miR-145-transfected CEPCs (Fig. 4B). The epithelium was degraded, thin (5.6±1.3 layers) and loosened with reduced cell density. Few cuboidal cells were found in the basal layer and cells were tends to be flatten or squamous in shape. The epithelium generated from miR-143-transfected CEPCs had morphology and compactness intermediate between control and miR-145 epithelia (8.3±1.6 layers) (Fig. 4C). The same results were obtained in duplicated experiments.

### Transcriptional regulation by miR-145

**Figure 5:**
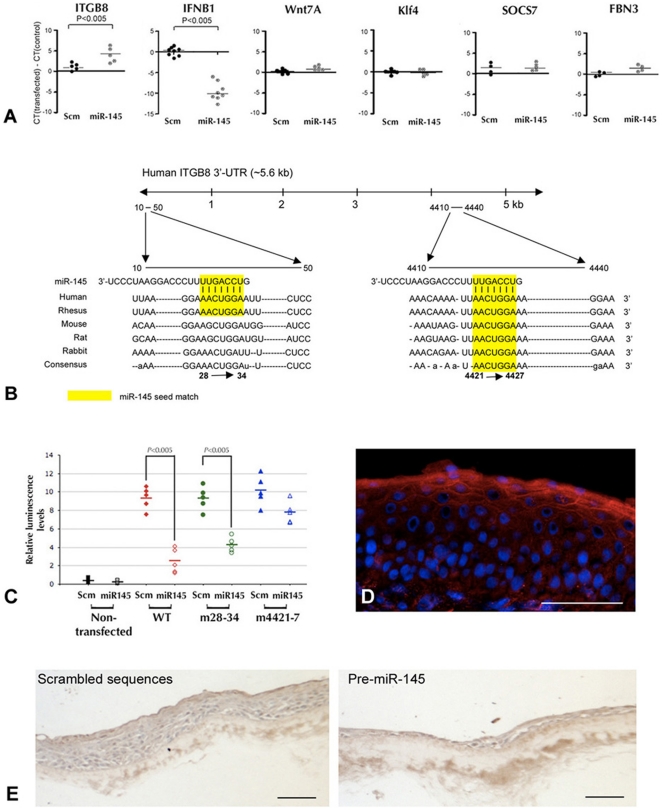
Target gene identification of miR-145 in human corneal epithelium. (A) Gene expression analysis by qPCR showing that ITGB8 was significantly down-regulated (*P* = 0.00024, paired Student's t-test) and IFNB1 was induced after miR-145 transfection (*P*<0.005). Wnt7A, Klf4, SOCS7 and FBN3 showed no changes. The dots represented ΔCT values (CT of transfected cells subtracted with CT of control cells). Horizontal lines indicated mean CT values. Scm: scrambled sequences. (B) Sequences of two miR-145 binding sites located in human ITGB8 3′UTR. Yellow shaded regions represent the conserved complementary nucleotides of miR-145 seed sequence in different species. The first 28–34^th^ nucleotide region is only conserved in primates whereas 4421–4427^th^ region is conserved in primates and rodents. (C) HeLa cells co-transfected with wildtype (WT) pCHECK-ITGB8_3′UTR and pre-miR-145 showed lower luciferase reporter activities when compared with cells transfected with scrambled sequences (n = 5) (red labels). Disruption of binding site 4421–4427^th^ region resulted in higher luciferase activity (blue labels) whereas mutation at 28–34^th^ region had no influence and the reduced luciferase activity levels (green labels) were similar as WT. * *P*<0.005, as compared to scrambled control (one-way ANOVA). (D) Immunofluorescence of ITGB8 in human limbal epithelium. Positive immunoreactivity was observed scattered in basal layer and continuous in superficial layers. No observable expression was noted in the parabasal layers. (E) Immunoperoxidase staining of ITGB8 in organotypic generated epithelia. Distinct ITGB8 expression was observed in epithelial layers generated from CEPCs transfected with scrambled sequences whereas reduced expression was found in epithelia from pre-miR-145-transfected CEPCs. Scale bars (D, E): 50 µm.

We compared the transcription profile of human corneal epithelial HCE cells transfected with lenti-miR-145 or with scrambled sequences using the Agilent Whole Human Genome Oligo Microarray platform, which screens for 41K human genes and transcripts. In two separate array experiments, miR-145 up-regulated 324 genes and down-regulated 277 genes by a five-fold difference compared to cells transfected with scrambled sequences (Tables S3A–B). Significant Gene Ontology terms enriched in the deregulated gene sets represented immune response (*P*<10^−7^), process (*P*<10^−5^), regulation (*P*<10^−3^), inflammatory response (*P*<10^−4^), cell defence (*P*<10^−5^), cell apoptosis (*P*<10^−3^), cell differentiation (*P*<10^−3^) and development (*P*<10^−3^). In addition, differentially regulated genes with >2 folds difference could be associated with epithelium development, cell proliferation and differentiation (Table 2, a full list is shown in Table S4). Real-time PCR analysis in 4 transfection experiments consistently showed that miR-145 markedly down-regulated integrin β8, ITGB8 (*P* = 0.00024, paired Student's *t* test) and up-regulated interferon β1 (IFNB1) (*P*<0.005) but not other candidate genes, such as Wnt7A, SOCS7 and Klf4 (Fig. 5A). Similar reduction of ITGB8 expression was observed in primary human CEPCs transfected with miR-145 (Fig. 3M). Predicted by TargetScan Human version 5.1 (http://www.targetscan.org), 2 conserved sites for miR-145 binding: 28–34^th^ and 4421–4427^th^ was found in the 3′UTR, equivalent to 3043–3049^th^ and 7436–7442^nd^ of human ITGB8 (NM_002214) (Fig. 5B). Notably, the first site is conserved in primates only whereas the second is found among primates, rodents and avian. Hence, influence of miR-145 on ITGB8 expression could be species-specific. To confirm miR-145 regulated ITGB8 expression by direct targeting the 3′UTR, we cloned the full-length wildtype ITGB8 3′UTR fragment downstream of psiCHECK-2 luciferase reporter gene and introduced mutated miR-145 target sites: 28–34^th^ and 4421–4427^th^, by site-directed mutagenesis. Luciferase expression, which represented promoter activity, was examined in HeLa cells co-transfected with the vectors and pre-miR-145 or scrambled sequences. The cells co-transfected with wildtype 3′UTR and miR-145 had reduced luminescence when compared to cells with wildtype 3′UTR and scrambled sequences (*P*<0.005, one-way ANOVA) (Fig. 5C). Reduced luminescence was also found in cells co-transfected with miR-145 and mutated 3′UTR at site 28–34^th^ but not with mutated site at 4421–4427^th^. Immunofluorescence showed membranous staining of ITGB8 in human limbal epithelium, particularly the basement membrane in contact with basal cells and superficial cell layers (Fig. 5D). Negative staining of the parabasal layers was coincident with positive miR-145 expression as shown by *in situ* hybridization (Figs. 2E and F). ITGB8 was strongly detected in cultured epithelia generated from CEPCs transfected with scrambled sequences but mild in those from pre-miR-145-transfected CEPCs (Fig. 5E).

**Table 2:** Selected human gene/transcript changes in miR-145 transfected HCE cells, compared to scrambled sequences.

Genes/transcripts	Fold changes	Reported features with cornea
***Upregulated, compared to scrambled control***		
- Interleukin 28B [IL28B]	100.5	Viral infection [63]
- Interferon β1 [IFNB1]	55.8	Infection, viral inflammation [64]
- Interferon α-inducible protein 6 [IFI6]	40.3	No information
- Endothelin converting enzyme 2 [ECE2]	7.0	Vasoconstriction [65]
- Complement component 1, q subcomponent-like 2 [C1QL2]	6.3	Inflammation
- Tumor necrosis factor α-induced protein 6 [TNFAIP6]	4.0	Angiogenesis, apoptosis, inflammation [66]
- Somatostatin receptor 4 [SSTR4]	3.9	Angiogenesis, inflammation [67]
- Retinoic acid receptor α [RARA]	2.2	Differentiation, inflammation [68]
***Downregulated, compared to scrambled control***		
- Angiopoietin 4 [ANGPT4]	0.08	Corneal angiogenesis, wound healing [69]
- Prostate stem cell antigen [PSCA]	0.11	Cornea development [70]
- Katanin p60 subunit A-like 2 [KARNAL2]	0.19	Cornea burn wounding [71]
- Cadherin 16 [CDH16]	0.26	Corneal limbal cell marker [72]
- Integrin β8 [ITGB8]	0.4	Corneal basal cell expression [73]
- Wingless-type MMTV integration site family 7A [WNT7A]	0.43	Corneal cell proliferation and wound closure [74]
- Suppressor of cytokine signalling 7 [SOCS7]	0.45	Corneal ulceration

## Discussion

In this study, we found differential expressions of 18 microRNAs between human limbal-peripheral and central corneal epithelia. Among them, miR-143 and miR-145 were expressed predominantly in the limbal epithelium but at very low levels in the central corneal epithelium. In primary human CEPCs, transfection of miR-145 up-regulated cytokeratin-3/12 and connexin-43 and concomitantly suppressed p63 and ABCG2 expression. We hypothesized that miR-145 could be an important regulatory molecule for human corneal epithelial differentiation. These cells developed to thinner and defective epithelium *in vitro*. This morphological alteration could be caused by miR-145 via the direct targeting on ITGB8. Meanwhile, disruption of binding site in ITGB8 3′UTR by site-directed mutagenesis eradicated the inhibition caused by miR-145. To our knowledge, this is the first report of microRNA regulation on human ocular cell differentiation and an example that microRNA can interfere with tissue development.

Located in human chromosome 5 and rodent chromosome 18, miR-143/145 are co-transcribed as one microRNA cluster from the same microRNA precursor. In a mouse model, both are initially expressed in the developing embryonic heart, followed by migration to smooth muscle cells of the aorta, intersomitic arteries, esophagus, lung, colon bladder and umbilical cord at later stages [24]. In adult mice, they are expressed in lung, skeletal muscle, heart and skin, and most abundantly in aorta and fat. MiR-145 is faintly expressed in self-renewing human ES cells but up-regulated during differentiation, indicating an inductive role on ES cell differentiation [25]. It directly targets on core plurpotency factors, repressing Oct4, Sox2 and Klf4, which are crucial to maintain the self-renewal and pluripotency capacity of ES cells and promote developments of the mesoderm and ectoderm lineages [24], [26]–[28]. A feedback mechanism has been proposed between miR-145 and Oct4 regulation [25]. Oct4 down-regulates miR-145 expression through repressive binding to its promoter. Hence, in ES cells, high Oct4 level suppresses miR-145 and the cells are capable of self-renewing. When entering into specific lineages, the differentiated cells express miR-145, which promotes differentiation and targets on Oct4 to suppress the self-renewal capability. In this study, miR-143/145 were expressed in the limbal epithelium, in particular the parabasal wing cell layers, but not in the central corneal and basal limbal epithelia. The parabasal region is enriched with proliferative TA cells with no self-renewal ability. The absence of Oct4 facilitates miR-145 expression. Furthermore, in normal adult CEPCs, Oct4, Sox2 and Klf4, as anticipated, are not detectable (Fig. 5A). This restricted the proof-of-concept study of miR-145 targets by knock-down experiments. Instead, over-expressing miR-145 in human CEPCs promoted CK3/12 and Cnx43 expression, which indicated the onset of corneal epithelial differentiation. Concurrently, these cells had reduced ABCG2 and p63 expressions, indicating their exit from stem cell proliferation state. As shown by *in vitro* corneal epithelium organotypic assay, a thinner and loosened epithelium was generated from miR-145-transfected human CEPCs. These findings strongly indicated that miR-145 suppressed the progenitor cell pool, and the cells were prone to differentiate, resulting in an underdeveloped epithelium with fewer cuboidal basal cells. We have attempted to transfect primary CEPCs by lenti-miR plasmid and observed about 70–80% transfection efficiency according to live GFP expression. We selected these cells for organotypic culture, which had demonstrated the influence of miR-145 on corneal epithelium development. Together with the reduced ITGB8 expression in the resulting epithelium, this likely showed that some cells could maintain miR-145 expression after culture for a month. However, we could not determine if this was due to transfection or endogenous expression in cells. Further experiments using lentivirus to obtain a long-term over-expression of miR-145 or specific miR-145 knockdown will be carried out to study the biological mechanism how miR-145 regulates corneal cell proliferation, migration and differentiation. Previous study using a zebrafish platform illustrated that miR-145 knockdown resulted in underdeveloped gut and heart [29].

We observed miR-145 down-regulated ITGB8 in human corneal epithelial cells and this might influence epithelium development and formation. In human limbal epithelium, ITGB8 was predominantly detectable in cell-cell boundary of superficial layers and also found scattered between cells in the basal layer. Interestingly, it was negligibly expressed in the parabasal region where miR-145 was detected. ITGB8, with its binding partner αV, is expressed in normal epithelial and neuronal cells *in vivo* and regulates transforming growth factor β (TGFβ) activation in various events, including cell growth, matrix modeling, epithelial-mesenchymal homeostasis, immune regulation and vasculogenesis [30]. Binding of Sp1, Sp3 and AP-1 transcription factors to its core promoter regulates ITGB8 expression in a p38-dependent manner [31]. TGFβactivation could lead to autocrine and paracrine signaling on cell growth and matrix production, which are important for epithelial cell adhesion and motility [32]. β8 interaction with Rho guanine nucleotide dissociation inhibitor-1 selectively stimulates Rac1, which regulates actin cytoskeleton arrangement, an important event in cell proliferation and differentiation [33], [34]. Also, αVβ8 integrin facilitates Fas induction [35], which is crucial for cell migration, production of inflammatory cytokines and corneal wound healing.

In addition, miR-145-transfected HCE cells had up-regulated IFNB1, which is known with anti-inflammatory activity. The cornea is described as “immune privilege”, characterized by suppression of systemic immunity after infection. This can be associated with low vascularization, presence of Fas ligand, which is a target of αVβ8 integrin, and TRAIL molecules and inhibitory substances in the aqueous humor [36]. As a consequence, corneal allografts usually survive longer than allografts in other body parts [37]. The immunologically protective mechanism in cornea can be associated with the production of nitric oxide, which intoxicates various pathogens [38]. Production of interleukins, interferon and TGFβ via αVβ8 integrin pathway) are pathogen-induced immunologically protective mechanism in corneal cells [39], [40]. Up-regulation of IFNB1 may contribute to enhancement of anti-inflammatory capability of corneal cells.

In conclusion, we discovered differential expression of microRNAs in human limbal and corneal epithelia. MiR-145 could be an important regulatory molecule for human corneal epithelial progenitor cell proliferation and differentiation. It is also crucial for the integrity of corneal epithelium, likely via ITGB8 targeting, which will be further investigated with *ITGB8* knockdown mice. Our findings provide the first identification of microRNAs expressed in adult tissue-specific site with regulatory effect on tissue cell differentiation.

## Supporting Information

Figure S1 (**A**) A schematic diagram illustrating the sample collection. LPC and CC epithelia separated by 1-mm (by width] uncut region were dissected out. (**B**) Clonal assay of CC and LPC isolated cells in culture for 7 days. Scale bar: 150 µm. (**C**) qPCR analysis to show ABCG2 expression in LPC but undetectable in LPC samples. (**D**) Western blotting of ABCG2 to validate the presence of CEPCs in LPC but not CC epithelia. Constant expression of p63α and β-actin was noted in LPC, CC and CJ (conjunctival epithelium).
(JPG)

Figure S2 Immunofluorescence of corneal progenitor cell and differentiation markers to validate the presence of CEPCs in human LPC compared to CC epithelia. Scale bars: 50 µm.
(JPG)

Figure S3 Expression analysis showing similar miR-16 (n = 7), miR-182 (n = 11) and miR-204 (n = 11) in LPC and CC epithelia.
(JPG)

Table S1 List of miRNA-specific primer in the miRNA expression analysis.
(DOC)

Table S2 Specific primers used in qPCR analysis.
(DOC)

Table S3 Human gene/transcript changes in miR-145 transfected HCE cells, compared to scrambled sequences.
(DOC)

Table S4 Significant Gene Ontology (GO) terms enriched in differential expressed gene list of miR-145- versus scrambled sequence-transfected cells (fold change ≥5).
(DOC)
