# Supplementary figures and images for: MicroRNA-145 Regulates Human Corneal Epithelial Differentiation
Source: PLoS One. 2011 Jun 20;6(6):e21249. doi: 10.1371/journal.pone.0021249 (PMC3119052; doi:10.1371/journal.pone.0021249)

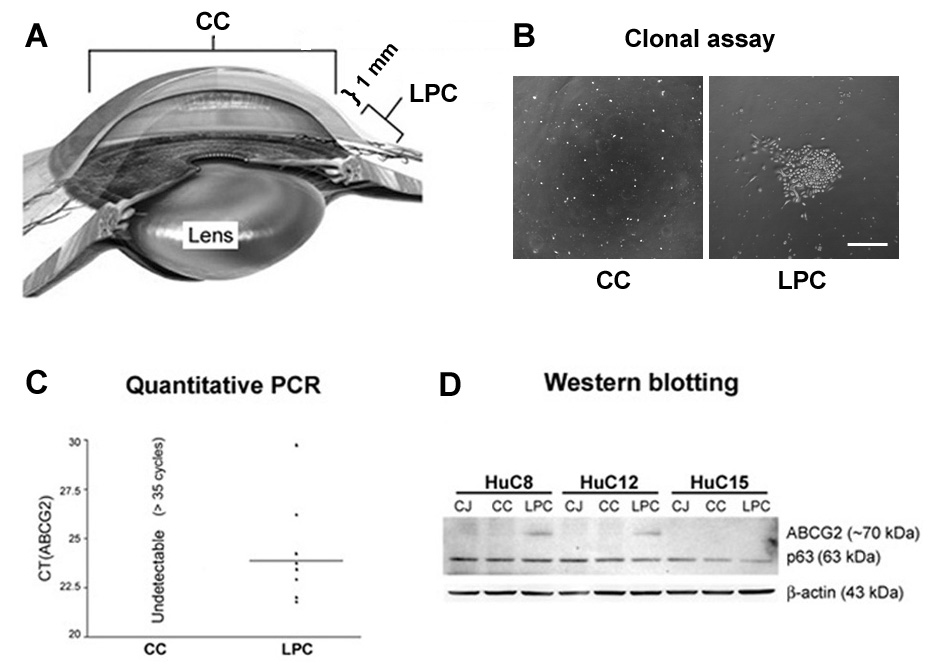

Supplement: Figure S1 — (A) A schematic diagram illustrating the sample collection. LPC and CC epithelia separated by 1-mm (by width] uncut region were dissected out. (B) Clonal assay of CC and LPC isolated cells in culture for 7 days. Scale bar: 150 µm. (C) qPCR analysis to show ABCG2 expression in LPC but undetectable in LPC samples. (D) Western blotting of ABCG2 to validate the presence of CEPCs in LPC but not CC epithelia. Constant expression of p63α and β-actin was noted in LPC, CC and CJ (conjunctival epithelium). (JPG) [file pone.0021249.s001.jpg]

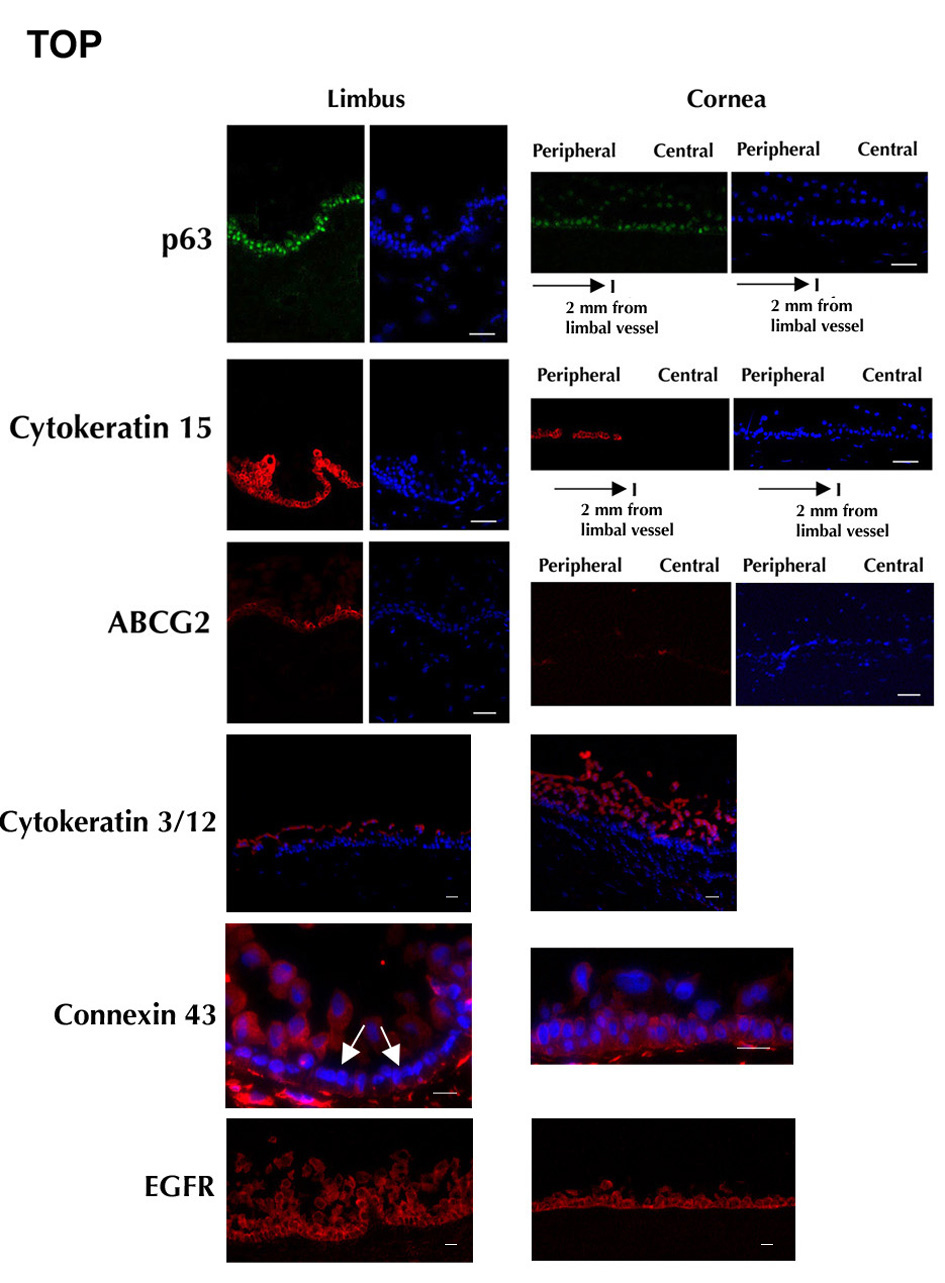

Supplement: Figure S2 — Immunofluorescence of corneal progenitor cell and differentiation markers to validate the presence of CEPCs in human LPC compared to CC epithelia. Scale bars: 50 µm. (JPG) [file pone.0021249.s002.jpg]

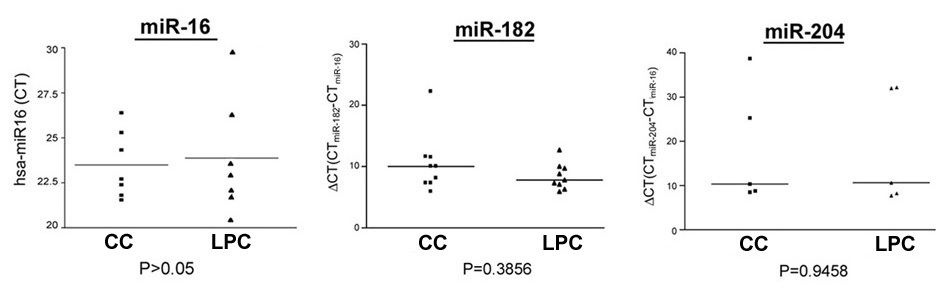

Supplement: Figure S3 — Expression analysis showing similar miR-16 (n = 7), miR-182 (n = 11) and miR-204 (n = 11) in LPC and CC epithelia. (JPG) [file pone.0021249.s003.jpg]
