# Supplementary material for: MicroRNA-145 Regulates Human Corneal Epithelial Differentiation
Source: PLoS One. 2011 Jun 20;6(6):e21249. doi: 10.1371/journal.pone.0021249 (PMC3119052; doi:10.1371/journal.pone.0021249)
Supplement: Table S1 — List of miRNA-specific primer in the miRNA expression analysis. (DOC) [file pone.0021249.s004.doc]

**Table S1. List of miRNA-specific primer in the miRNA expression analysis**

| **MicroRNAs** | **Target Sequences** (**5’-3’]** |
| --- | --- |
| hsa-let-7a | UGAGGUAGUAGGUUGUAUAGUU |
| hsa-miR-16 | UAGCAGCACGUAAAUAUUGGCG |
| hsa-miR-21 | UAGCUUAUCAGACUGAUGUUGA |
| hsa-miR-26a | UUCAAGUAAUUCAGGAUAGGUU |
| hsa-miR-143 | UGAGAUGAAGCACUGUAGCUC |
| hsa-miR-145 | GUCCAGUUUUCCCAGGAAUCCCU |
| hsa-miR-182 | UUUGGCAAUGGUAGAACUCACA |
| hsa-miR-184 | UGGACGGAGAACUGAUAAGGGU |
| hsa-miR-204 | UUCCCUUUGUCAUCCUAUGCCU |
| hsa-miR-302a | UAAGUGCUUCCAUGUUUUGGUG |
| hsa-miR-302d | UAAGUGCUUCCAUGUUUGAGUGU |
| hsa-miR-320 | AAAAGCUGGGUUGAGAGGGCGAA |
| hsa-miR-338 | UCCAGCAUCAGUGAUUUUGUUGA |
| hsa-miR-371 | GUGCCGCCAUCUUUUGAGUGU |
| hsa-miR-372 | AAAGUGCUGCGACAUUUGAGCGU |
| hsa-miR-373 | GAAGUGCUUCGAUUUUGGGGUGU |
| hsa-miR-373# | ACUCAAAAUGGGGGCGCUUUCC |
