# Supplementary material for: MicroRNA-145 Regulates Human Corneal Epithelial Differentiation
Source: PLoS One. 2011 Jun 20;6(6):e21249. doi: 10.1371/journal.pone.0021249 (PMC3119052; doi:10.1371/journal.pone.0021249)
Supplement: Table S2 — Specific primers used in qPCR analysis. (DOC) [file pone.0021249.s005.doc]

**Table S2. Specific primers used in qPCR analysis**

| **Genes** | **GeneBank No.** |  | **Sequence (5’-3’)** | **Product (bp)** |
| --- | --- | --- | --- | --- |
| Angiopoietin 4 [ANGPT4] | NM_015985 | F  R | GTGGTGTCTACACCATCCAGGT  TCCGCTGAAAATTCACGGTGCC | 129 |
| Annexin A13 [ANXA13] | NM_004306 | F  R | GCGTTGGCCCTTCTGGACCG  CAGCGGCCTTCCCCTGCATC | 320 |
| ATP-binding cassette, sub-family G, member 2 [ABCG2] | NM_004827 | F  R | AGCGCACGCATCCTGAGAT  GCCTTCAGGTCATTGGAAGCT | 164 |
| Cadherin 16 [CDH16] | NM_004062 | F  R | CGGTGCTTGTGGAGGCCCAG  CTTGGCGGGGTGTGCAGAGG | 144 |
| Complement component 1, q subcomponent-like 2 [C1QL2] | NM_182528 | F  R | TCACCTACCACATCCTCATGCG  CGCTGTTACTGGCGTAGTCGTA | 132 |
| Endothelin converting enzyme 2 [ECE2] | NM_032331 | F  R | GGAACAGTGCCCTGAGCTAC  CTTCTCGAGCACCACATCAA | 188 |
| Fibrillin 3 [FBN3] | NM_000138 | F  R | ATGGGCTTCGTGCCCAGTGC  TGCAGTTCCCGCCGCTTCTG | 160 |
| Integrin, β8 [ITGB8] | NM_002214 | F  R | TCCCGCCTCGTTCCTCTGGG  CTTCGGCTCCTGGACGCAGC | 332 |
| Interferon, β1 [IFNB1] | NM_002176 | F  R | GCATTACCTGAAGGCCAAGG  GCAATTGTCCAGTCCCAGAG | 159 |
| Interferon regulatory factor 7 [IRF7] | NM_001572 | F  R | GTGCAAGAGCCCAGCCCAGG  CGGCACAGCCAGGGTTCCAG | 536 |
| Katanin p60 subunit A-like 2 [KATNAL2] | NM_031303 | F  R | CGCCTGGAAAATGCCAACTTCG  CCTTGATGGCATCTGTGAGCAG | 127 |
| p63α | NM_003722 | F  R | CAGCATGAACAAGCTGCCTTCT  GGGTGGGGCTGAGTCCAT | 158 |
| Prostate stem cell antigen [PSCA] | NM_005672 | F  R | TGCTGTGCTACTCCTGCAAAGC  GAGTCATCCACGCAGTTCAAGC | 100 |
| Retinoic acid receptor, α  | NM_000964 | F  R | GGGAATCCTGAATCGAGCTG  AAAGATGCCACTCCTAGATGGG | 142 |
| Somatostatin receptor 4 [SSTR4] | NM_001052 | F  R | CTATGGCTTCCTCTCCGACAAC  GCTCTTGAGAGCAGTGGCATAG | 130 |
| Suppressor of cytokine signaling 7 [SOCS7] | NM_014598 | F  R | TACCTCCGGTGCCCTTCCCG  GAGGTCAGGCCCCGCTGAGA | 430 |
| Tumor necrosis factor, α-induced protein 6 [TNFAIP6] | NM_007115 | F  R | ACCACAGAGAAGCACGGTCT  CAACTCTGCCCTTAGCCATC | 165 |
| Wingless-type MMTV integration site family, member 7A  [Wnt7A] | NM_004625 | F  R | CTGGAACTGCTCTGCACTGG  GGTGGTACTGGCCTTGCTTC | 185 |
