# Supplementary material for: MicroRNA-145 Regulates Human Corneal Epithelial Differentiation
Source: PLoS One. 2011 Jun 20;6(6):e21249. doi: 10.1371/journal.pone.0021249 (PMC3119052; doi:10.1371/journal.pone.0021249)
Supplement: Table S3 — Human gene/transcript changes in miR-145 transfected HCE cells, compared to scrambled sequences. (DOC) [file pone.0021249.s006.doc]

**Table S3. Human gene / transcript changes in miR-145 transfected HCE cells, compared to scrambled sequences**

***A. Candidate genes up-regulated by 5 folds in miR-145 transfection***

|  | **Gene Symbols** | **Fold changes** | **Description** |
| --- | --- | --- | --- |
| 1 | CARD18 | 283.81 | Homo sapiens caspase recruitment domain family, member 18, mRNA [NM_021571] |
| 2 | SOX2 | 208.75 | Homo sapiens SRY (sex determining region Y)-box 2, mRNA [NM_003106] |
| 3 | NADSYN1 | 101.83 | Homo sapiens NAD synthetase 1, mRNA [NM_018161] |
| 4 | IL28B | 100.46 | Homo sapiens interleukin 28B (interferon, lambda 3), mRNA [NM_172139] |
| 5 | IL28A | 94.13 | Homo sapiens interleukin 28A (interferon, lambda 2), mRNA [NM_172138] |
| 6 | CXCL10 | 72.84 | Homo sapiens chemokine (C-X-C motif) ligand 10, mRNA [NM_001565] |
| 7 | CSF2RA | 70.58 | Homo sapiens colony stimulating factor 2 receptor, alpha, low-affinity, mRNA [NM_172249] |
| 8 | CCL3L3 | 67.98 | Homo sapiens chemokine (C-C motif) ligand 3-like 3, mRNA [NM_001001437] |
| 9 | CXCL11 | 67.22 | Homo sapiens chemokine (C-X-C motif) ligand 11, mRNA [NM_005409] |
| 10 | EFS | 64.68 | Homo sapiens embryonal Fyn-associated substrate, mRNA [NM_005864] |
| 11 | TRIM22 | 58.87 | Homo sapiens tripartite motif-containing 22, mRNA [NM_006074] |
| 12 | TRIM9 | 58.57 | Homo sapiens tripartite motif-containing 9, transcript variant 1, mRNA [NM_015163] |
| 13 | NPTX2 | 56.84 | Homo sapiens neuronal pentraxin II, mRNA [NM_002523] |
| 14 | IFNB1 | 55.79 | Homo sapiens interferon, beta 1, mRNA [NM_002176] |
| 15 | MX2 | 51.98 | Homo sapiens myxovirus resistance 2, mRNA [NM_002463] |
| 16 | NLGN4Y | 50.61 | Homo sapiens neuroligin 4, Y-linked, transcript variant 1, mRNA [NM_014893] |
| 17 | GREM2 | 41.79 | Homo sapiens gremlin 2, cysteine knot superfamily, mRNA [NM_022469] |
| 18 | CSN2 | 41.13 | Homo sapiens casein beta, mRNA [NM_001891] |
| 19 | TRIM22 | 40.85 | Homo sapiens tripartite motif-containing 22, mRNA [NM_006074] |
| 20 | IFI6 | 40.34 | Homo sapiens interferon, alpha-inducible protein 6, transcript variant 3, mRNA [NM_022873] |
| 21 | SLC9A11 | 39.59 | Homo sapiens solute carrier family 9, member 11, mRNA [NM_178527] |
| 22 | CXCL11 | 39.03 | Homo sapiens chemokine (C-X-C motif) ligand 11, mRNA [NM_005409] |
| 23 | IFI44L | 38.76 | Homo sapiens interferon-induced protein 44-like, mRNA [NM_006820] |
| 24 | CALN1 | 37.68 | Homo sapiens calneuron 1, transcript variant 1, mRNA [NM_031468] |
| 25 | IL29 | 36.19 | Homo sapiens interleukin 29, mRNA [NM_172140] |
| 26 | KLK13 | 35.45 | Homo sapiens kallikrein-related peptidase 13, mRNA [NM_015596] |
| 27 | OAS2 | 33.68 | Homo sapiens 2'-5'-oligoadenylate synthetase 2, transcript variant 1, mRNA [NM_016817] |
| 28 | MX2 | 33.01 | Homo sapiens myxovirus resistance 2, mRNA [NM_002463] |
| 29 | KIR3DX1 | 32.01 | Homo sapiens killer cell immunoglobulin-like receptor, three domains, X1, mRNA. [BC033195] |
| 30 | CCL3L3 | 29.73 | Homo sapiens chemokine (C-C motif) ligand 3-like 3, mRNA [NM_001001437] |
| 31 | SPINK5 | 29.57 | Homo sapiens serine peptidase inhibitor, Kazal type 5, mRNA [NM_006846] |
| 32 | OAS1 | 28.99 | Homo sapiens 2',5'-oligoadenylate synthetase 1, mRNA [NM_002534] |
| 33 | LGALS9C | 27.95 | Homo sapiens lectin, galactoside-binding, soluble, 9C, mRNA [NM_001040078] |
| 34 | CHDH | 27.73 | Homo sapiens choline dehydrogenase (CHDH), mRNA [NM_018397] |
| 35 | CDR1 | 26.61 | Homo sapiens cerebellar degeneration-related protein 1, mRNA [NM_004065] |
| 36 | SLC7A13 | 26.36 | Homo sapiens solute carrier family 7, member 13, mRNA [NM_138817] |
| 37 | OAS2 | 26.08 | Homo sapiens 2'-5'-oligoadenylate synthetase 2, transcript variant 1, mRNA [NM_016817] |
| 38 | PURG | 24.92 | Homo sapiens purine-rich element binding protein G, mRNA [NM_001015508] |
| 39 | FAM135B | 24.79 | Homo sapiens family with sequence similarity 135, member B, mRNA [NM_015912] |
| 40 | ZNF483 | 23.11 | Homo sapiens zinc finger protein 483, transcript variant 1, mRNA [NM_133464] |
| 41 | RSAD2 | 23.06 | Homo sapiens radical S-adenosyl methionine domain containing 2, mRNA [NM_080657] |
| 42 | PADI4 | 22.51 | Homo sapiens peptidyl arginine deiminase, type IV, mRNA [NM_012387] |
| 43 | PSD | 21.58 | Homo sapiens pleckstrin and Sec7 domain containing, mRNA [NM_002779] |
| 44 | PODN | 21.58 | Homo sapiens podocan, mRNA [NM_153703] |
| 45 | IL5RA | 21.45 | Homo sapiens interleukin 5 receptor, alpha, transcript variant 1, mRNA [NM_000564] |
| 46 | APOB48R | 21.28 | Homo sapiens apolipoprotein B48 receptor (APOB48R), mRNA [NM_018690] |
| 47 | GPR125 | 20.87 | Homo sapiens G protein-coupled receptor 125, mRNA [BC026009] |
| 48 | HOXD13 | 20.28 | Homo sapiens homeobox D13, mRNA [NM_000523] |
| 49 | TMEM140 | 19.98 | Homo sapiens transmembrane protein 140, mRNA [NM_018295] |
| 50 | RGS1 | 19.90 | Homo sapiens regulator of G-protein signaling 1, mRNA [NM_002922] |
| 51 | SELENBP1 | 19.63 | Homo sapiens selenium binding protein 1 (SELENBP1), mRNA [NM_003944] |
| 52 | LRFN1 | 19.10 | Homo sapiens leucine rich repeat and fibronectin type III domain containing 1, mRNA [BC014678] |
| 53 | MMP13 | 19.04 | Homo sapiens matrix metallopeptidase 13 (collagenase 3), mRNA [NM_002427] |
| 54 | MX1 | 18.70 | Homo sapiens myxovirus resistance 1, interferon-inducible protein p78, mRNA [NM_002462] |
| 55 | ATP10A | 18.38 | Homo sapiens ATPase, class V, type 10A, mRNA [NM_024490] |
| 56 | KCNT1 | 18.00 | Homo sapiens potassium channel, subfamily T, member 1, mRNA [NM_020822] |
| 57 | HRASLS2 | 17.98 | Homo sapiens HRAS-like suppressor 2, mRNA [NM_017878] |
| 58 | EPSTI1 | 17.18 | Homo sapiens epithelial stromal interaction 1, transcript variant 2, mRNA [NM_033255] |
| 59 | SCN2B | 16.21 | Homo sapiens sodium channel, voltage-gated, type II, beta, mRNA [NM_004588] |
| 60 | USP18 | 15.90 | Homo sapiens ubiquitin specific peptidase 18, mRNA [NM_017414] |
| 61 | UMODL1 | 15.89 | Homo sapiens uromodulin-like 1, transcript variant 2, mRNA [NM_173568] |
| 62 | GBP5 | 15.88 | Homo sapiens guanylate binding protein 5, transcript variant 1, mRNA [NM_052942] |
| 63 | LANCL2 | 15.42 | Homo sapiens LanC lantibiotic synthetase component C-like 2, mRNA [NM_018697] |
| 64 | CCL5 | 15.34 | Homo sapiens chemokine (C-C motif) ligand 5, mRNA [NM_002985] |
| 65 | F2 | 15.19 | Homo sapiens coagulation factor II, mRNA [NM_000506] |
| 66 | PCDH11Y | 14.96 | Homo sapiens protocadherin 11 Y-linked, transcript variant c, mRNA [NM_032973] |
| 67 | USP18 | 14.84 | Homo sapiens ubiquitin specific peptidase 18, mRNA [NM_017414] |
| 68 | PRSS21 | 14.80 | Homo sapiens protease, serine, 21, transcript variant 1, mRNA [NM_006799] |
| 69 | FAM46D | 14.76 | Homo sapiens family with sequence similarity 46, member D, mRNA [NM_152630] |
| 70 | TUBB1 | 13.91 | Homo sapiens tubulin, beta 1, mRNA [NM_030773] |
| 71 | HSH2D | 13.54 | Homo sapiens hematopoietic SH2 domain containing, mRNA [NM_032855] |
| 72 | RSAD2 | 12.97 | Homo sapiens radical S-adenosyl methionine domain containing 2, mRNA [NM_080657] |
| 73 | XAF1 | 12.89 | Homo sapiens XIAP associated factor 1, transcript variant 1, mRNA [NM_017523] |
| 74 | CSAG2 | 12.81 | Homo sapiens CSAG family, member 2, transcript variant 2, mRNA [NM_004909] |
| 75 | LAMP3 | 12.71 | Homo sapiens lysosomal-associated membrane protein 3, mRNA [NM_014398] |
| 76 | MYH6 | 12.51 | Homo sapiens myosin, heavy chain 6, alpha, mRNA [NM_002471] |
| 77 | CCDC54 | 12.51 | Homo sapiens coiled-coil domain containing 54, mRNA [NM_032600] |
| 78 | CTSS | 12.24 | Homo sapiens cathepsin S, mRNA [NM_004079] |
| 79 | TNFSF10 | 12.20 | Homo sapiens tumor necrosis factor (ligand) superfamily, member 10, mRNA [NM_003810] |
| 80 | TM7SF4 | 12.19 | Homo sapiens transmembrane 7 superfamily member 4, mRNA [NM_030788] |
| 81 | CSAG1 | 12.01 | Homo sapiens chondrosarcoma associated gene 1, mRNA [NM_153478] |
| 82 | LGALS9 | 12.01 | Homo sapiens lectin, galactoside-binding, soluble, 9, mRNA [NM_009587] |
| 83 | CYP46A1 | 11.90 | Homo sapiens cytochrome P450, family 46A, polypeptide 1, mRNA [NM_006668] |
| 84 | CSAG2 | 11.88 | Homo sapiens CSAG family, member 2, transcript variant 1, mRNA [NM_001080848] |
| 85 | PRAMEF8 | 11.72 | Homo sapiens PRAME family member 8, mRNA [NM_001012276] |
| 86 | PIWIL2 | 11.69 | Homo sapiens piwi-like 2, transcript variant 2, mRNA [NM_018068] |
| 87 | HRASLS2 | 11.53 | Homo sapiens HRAS-like suppressor 2, mRNA [NM_017878] |
| 88 | RANBP3 | 11.52 | Homo sapiens RanBP3, splice variant, mRNA [Y08699] |
| 89 | CD38 | 11.39 | Homo sapiens CD38 molecule, mRNA [NM_001775] |
| 90 | GPR109B | 11.36 | Homo sapiens G protein-coupled receptor 109B, mRNA [NM_006018] |
| 91 | GLT1D1 | 11.14 | Homo sapiens glycosyltransferase 1 domain containing 1, mRNA [NM_144669] |
| 92 | IFITM1 | 11.11 | Homo sapiens interferon induced transmembrane protein 1, mRNA [NM_003641] |
| 93 | ITLN1 | 11.10 | Homo sapiens intelectin 1, mRNA [NM_017625] |
| 94 | ETV7 | 10.96 | Homo sapiens ets variant 7, mRNA [NM_016135] |
| 95 | IDO1 | 10.89 | Homo sapiens indoleamine 2,3-dioxygenase 1, mRNA [NM_002164] |
| 96 | SNTB1 | 10.83 | Homo sapiens Tax interaction protein 43 mRNA, partial cds. [AF028828] |
| 97 | ITSN1 | 10.80 | Intersectin 1 (SH3 domain protein)[ENST00000381318] |
| 98 | CDH5 | 10.68 | Homo sapiens cadherin 5, type 2, mRNA [NM_001795] |
| 99 | CYCS | 10.57 | Homo sapiens cytochrome c, mRNA [NM_018947] |
| 100 | F7 | 10.43 | Homo sapiens coagulation factor VII, transcript variant 1, mRNA [NM_000131] |
| 101 | PROX1 | 10.18 | Homo sapiens prospero homeobox 1, mRNA [NM_002763] |
| 102 | SLC2A2 | 10.10 | Homo sapiens solute carrier family 2, member 2, mRNA [NM_000340] |
| 103 | VIT | 10.09 | Homo sapiens vitrin, mRNA [NM_053276] |
| 104 | HBE1 | 10.08 | Homo sapiens hemoglobin, epsilon 1, mRNA [NM_005330] |
| 105 | SLC15A3 | 10.07 | Homo sapiens solute carrier family 15, member 3, mRNA [NM_016582] |
| 106 | OLR1 | 10.07 | Homo sapiens oxidized low density lipoprotein receptor 1, mRNA [NM_002543] |
| 107 | KBTBD3 | 9.98 | Homo sapiens kelch repeat and BTB (POZ) domain containing 3, mRNA [NM_198439] |
| 108 | EPSTI1 | 9.95 | Epithelial stromal interaction 1 [ENST00000313624] |
| 109 | TAC3 | 9.78 | Homo sapiens tachykinin 3, mRNA [NM_013251] |
| 110 | CP | 9.75 | Homo sapiens ceruloplasmin, mRNA [NM_000096] |
| 111 | TMEM30B | 9.71 | Homo sapiens transmembrane protein 30B, mRNA [NM_001017970] |
| 112 | LRRTM2 | 9.65 | Homo sapiens leucine rich repeat transmembrane neuronal 2, mRNA [NM_015564] |
| 113 | COL2A1 | 9.62 | Homo sapiens collagen, type II, alpha 1, transcript variant 1, mRNA [NM_001844] |
| 114 | ZBTB32 | 9.56 | Homo sapiens zinc finger and BTB domain containing 32, mRNA [NM_014383] |
| 115 | ATP8B4 | 9.40 | Homo sapiens ATPase, class I, type 8B, member 4, mRNA [NM_024837] |
| 116 | LENEP | 9.37 | Homo sapiens lens epithelial protein, mRNA [NM_018655] |
| 117 | LGALS9C | 9.34 | Homo sapiens lectin, galactoside-binding, soluble, 9C, mRNA [NM_001040078] |
| 118 | SSTR5 | 9.34 | Homo sapiens somatostatin receptor 5, transcript variant 1, mRNA [NM_001053] |
| 119 | CFB | 9.31 | Homo sapiens complement factor B, mRNA [NM_001710] |
| 120 | PLAC9 | 9.27 | Placenta-specific 9 [ENST00000372263] |
| 121 | NLRP4 | 9.27 | Homo sapiens NLR family, pyrin domain containing 4, mRNA [NM_134444] |
| 122 | NPBWR2 | 9.19 | Homo sapiens neuropeptides B/W receptor 2, mRNA [NM_005286] |
| 123 | UBD | 9.17 | Homo sapiens ubiquitin D, mRNA [NM_006398] |
| 124 | RASGRP3 | 9.11 | Homo sapiens RAS guanyl releasing protein 3, transcript variant 2, mRNA [NM_170672] |
| 125 | TUSC5 | 9.09 | Homo sapiens tumor suppressor candidate 5, mRNA [NM_172367] |
| 126 | MYCT1 | 9.09 | Homo sapiens myc target 1, mRNA [NM_025107] |
| 127 | NKX2-1 | 9.08 | Homo sapiens NK2 homeobox 1, transcript variant 2, mRNA [NM_003317] |
| 128 | MEOX1 | 8.97 | Homo sapiens mesenchyme homeobox 1, transcript variant 1, mRNA [NM_004527] |
| 129 | KAAG1 | 8.93 | Homo sapiens kidney associated antigen 1, mRNA [NM_181337] |
| 130 | KNG1 | 8.90 | Homo sapiens kininogen 1, transcript variant 2, mRNA [NM_000893] |
| 131 | BATF2 | 8.83 | Homo sapiens basic leucine zipper transcription factor, ATF-like 2, mRNA [NM_138456] |
| 132 | ZNF683 | 8.77 | Homo sapiens zinc finger protein 683, transcript variant 2, mRNA [NM_173574] |
| 133 | CD8B | 8.74 | Homo sapiens CD8b molecule, transcript variant 4, mRNA [NM_172102] |
| 135 | IL1F6 | 8.67 | Homo sapiens interleukin 1 family, member 6, mRNA [NM_014440] |
| 136 | ISG15 | 8.65 | Homo sapiens ISG15 ubiquitin-like modifier, mRNA [NM_005101] |
| 137 | CYP4X1 | 8.65 | Homo sapiens cytochrome P450, family 4, subfamily X, polypeptide 1, mRNA [NM_178033] |
| 138 | ALOX15 | 8.64 | Homo sapiens arachidonate 15-lipoxygenase, mRNA [NM_001140] |
| 139 | MAP1LC3C | 8.47 | Homo sapiens microtubule-associated protein 1 light chain 3 gamma, mRNA [NM_001004343] |
| 140 | FCAR | 8.46 | Homo sapiens Fc fragment of IgA, receptor for, transcript variant 1, mRNA [NM_002000] |
| 141 | ARL10 | 8.40 | Homo sapiens ADP-ribosylation factor-like 10, mRNA [NM_173664] |
| 142 | GPR26 | 8.39 | Homo sapiens G protein-coupled receptor 26, mRNA [NM_153442] |
| 143 | IFI35 | 8.37 | Homo sapiens interferon-induced protein 35, mRNA [NM_005533] |
| 144 | UROC1 | 8.35 | Homo sapiens urocanase domain containing 1, transcript variant 1, mRNA [NM_144639] |
| 145 | DYNC1I1 | 8.30 | Homo sapiens dynein, cytoplasmic 1, intermediate chain 1, mRNA [NM_004411] |
| 146 | GBP1 | 8.29 | Homo sapiens guanylate binding protein 1, interferon-inducible, mRNA [NM_002053] |
| 147 | PGA3 | 8.29 | Homo sapiens pepsinogen 3, group I, mRNA [NM_001079807] |
| 148 | TMEM232 | 8.28 | Homo sapiens transmembrane protein 232, mRNA [NM_001039763] |
| 149 | CTNNA2 | 8.21 | Homo sapiens catenin (cadherin-associated protein), alpha 2, mRNA [NM_004389] |
| 150 | RPS4Y2 | 8.20 | Homo sapiens ribosomal protein S4, Y-linked 2, mRNA [NM_001039567] |
| 151 | OR8J1 | 8.19 | Homo sapiens olfactory receptor, family 8, subfamily J, member 1, mRNA [NM_001005205] |
| 152 | TMEM132B | 8.17 | Homo sapiens transmembrane protein 132B, mRNA [NM_052907] |
| 153 | BHLHE22 | 8.15 | Homo sapiens basic helix-loop-helix family, member e22, mRNA [NM_152414] |
| 154 | NHEDC1 | 8.13 | Homo sapiens Na+/H+ exchanger domain containing 1, mRNA [NM_139173] |
| 155 | PARP9 | 8.06 | Homo sapiens poly (ADP-ribose) polymerase family, member 9, mRNA [NM_031458] |
| 156 | OR51B2 | 8.02 | Homo sapiens olfactory receptor, family 51, subfamily B, member 2, mRNA [NM_033180] |
| 157 | GBP4 | 7.97 | Homo sapiens guanylate binding protein 4, mRNA [NM_052941] |
| 158 | SLC8A2 | 7.89 | Homo sapiens solute carrier family 8, member 2, mRNA [NM_015063] |
| 159 | CCL4 | 7.87 | Homo sapiens chemokine (C-C motif) ligand 4, transcript variant 1, mRNA [NM_002984] |
| 160 | ATRNL1 | 7.85 | Homo sapiens attractin-like 1, mRNA [NM_207303] |
| 161 | SLCO1A2 | 7.82 | Homo sapiens solute carrier organic anion transporter family, member 1A2, mRNA [NM_134431] |
| 162 | NPY5R | 7.81 | Homo sapiens neuropeptide Y receptor Y5, mRNA [NM_006174] |
| 163 | CGB1 | 7.75 | Homo sapiens chorionic gonadotropin, beta polypeptide 1, mRNA [NM_033377] |
| 164 | TRIOBP | 7.73 | Homo sapiens TRIO and F-actin binding protein, mRNA [NM_001039141] |
| 165 | CH25H | 7.72 | Homo sapiens cholesterol 25-hydroxylase, mRNA [NM_003956] |
| 166 | GFRA2 | 7.70 | Homo sapiens GDNF family receptor alpha 2, transcript variant 1, mRNA [NM_001495] |
| 167 | SH2D1A | 7.70 | Homo sapiens SH2 domain protein 1A, transcript variant 1, mRNA [NM_002351] |
| 168 | FTMT | 7.69 | Homo sapiens ferritin mitochondrial, mRNA [NM_177478] |
| 169 | GDF7 | 7.61 | Growth differentiation factor 7 [ENST00000272224] |
| 170 | ACTL7B | 7.58 | Homo sapiens actin-like 7B, mRNA [NM_006686] |
| 171 | NFAM1 | 7.55 | Homo sapiens NFAT activating protein with ITAM motif 1, mRNA [NM_145912] |
| 172 | CCL26 | 7.50 | Homo sapiens chemokine (C-C motif) ligand 26, mRNA [NM_006072] |
| 173 | SLC8A2 | 7.44 | Homo sapiens solute carrier family 8, member 2, mRNA [NM_015063] |
| 174 | SLC22A24 | 7.34 | Homo sapiens solute carrier family 22, member 24, mRNA [NM_001136506] |
| 175 | CYP2A7 | 7.32 | Homo sapiens cytochrome P450, family 2, subfamily A, polypeptide 7, mRNA [NM_000764] |
| 176 | GAS2L2 | 7.27 | Homo sapiens growth arrest-specific 2 like 2, mRNA [NM_139285] |
| 177 | KLRD1 | 7.27 | Homo sapiens killer cell lectin-like receptor subfamily D, member 1, mRNA [NM_002262] |
| 178 | TAAR6 | 7.26 | Homo sapiens trace amine associated receptor 6, mRNA [NM_175067] |
| 179 | NPR1 | 7.24 | Homo sapiens natriuretic peptide receptor A/guanylate cyclase A, mRNA [NM_000906] |
| 180 | MLANA | 7.14 | Homo sapiens melan-A, mRNA [NM_005511] |
| 181 | PHF16 | 7.13 | Homo sapiens PHD finger protein 16, transcript variant 1, mRNA [NM_014735] |
| 182 | BTK | 7.13 | Homo sapiens Bruton agammaglobulinemia tyrosine kinase, mRNA [NM_000061] |
| 183 | TMPRSS12 | 7.11 | Homo sapiens transmembrane protease, serine 12, mRNA [NM_182559] |
| 184 | KCNA5 | 7.11 | Homo sapiens potassium voltage-gated channel, member 5, mRNA [NM_002234] |
| 185 | CD160 | 7.09 | Homo sapiens CD160 molecule, mRNA [NM_007053] |
| 186 | SAMD9L | 7.06 | Homo sapiens sterile alpha motif domain containing 9-like, mRNA [NM_152703] |
| 187 | IFITM3 | 7.00 | Homo sapiens interferon induced transmembrane protein 3, mRNA [NM_021034] |
| 188 | ECE2 | 6.96 | Homo sapiens endothelin converting enzyme 2, transcript variant 1, mRNA [NM_014693] |
| 189 | RTP4 | 6.94 | Homo sapiens receptor (chemosensory) transporter protein 4, mRNA [NM_022147] |
| 190 | NLRP12 | 6.91 | Homo sapiens NLR family, pyrin domain containing 12, mRNA [NM_033297] |
| 191 | FTMT | 6.88 | Homo sapiens ferritin mitochondrial, mRNA [NM_177478] |
| 192 | PTCHD2 | 6.88 | Homo sapiens patched domain containing 2, mRNA [NM_020780] |
| 193 | GATA1 | 6.86 | Homo sapiens GATA binding protein 1, mRNA [NM_002049] |
| 194 | GBP1 | 6.85 | Homo sapiens guanylate binding protein 1, interferon-inducible, mRNA [NM_002053] |
| 195 | IFITM2 | 6.85 | Homo sapiens interferon induced transmembrane protein 2, mRNA [NM_006435] |
| 196 | SPARCL1 | 6.82 | Homo sapiens SPARC-like 1, transcript variant 2, mRNA [NM_004684] |
| 197 | CSF2RA | 6.80 | Homo sapiens colony stimulating factor 2 receptor, alpha, low-affinity, mRNA [NM_172247] |
| 198 | FAM131A | 6.75 | Homo sapiens family with sequence similarity 131, member A, mRNA [NM_144635] |
| 199 | FGA | 6.72 | Homo sapiens fibrinogen alpha chain, transcript variant alpha-E, mRNA [NM_000508] |
| 200 | GZMA | 6.72 | Homo sapiens granzyme A, mRNA [NM_006144] |
| 201 | TCF15 | 6.72 | Homo sapiens transcription factor 15 (basic helix-loop-helix), mRNA [NM_004609] |
| 202 | IFIT1 | 6.68 | Homo sapiens interferon-induced protein with tetratricopeptide repeats 1, mRNA [NM_001548] |
| 203 | GDF2 | 6.67 | Homo sapiens growth differentiation factor 2, mRNA [NM_016204] |
| 204 | PRND | 6.66 | Homo sapiens prion protein 2 (dublet), mRNA [NM_012409] |
| 205 | HIST4H4 | 6.64 | Homo sapiens histone cluster 4, H4, mRNA [NM_175054] |
| 206 | SLC15A2 | 6.61 | Homo sapiens solute carrier family 15, member 2, mRNA [NM_021082] |
| 207 | GBP4 | 6.61 | Homo sapiens guanylate binding protein 4, mRNA [NM_052941] |
| 208 | APOH | 6.60 | Homo sapiens apolipoprotein H, mRNA [NM_000042] |
| 209 | CCDC37 | 6.58 | Homo sapiens coiled-coil domain containing 37, mRNA [NM_182628] |
| 210 | ABCD2 | 6.55 | Homo sapiens ATP-binding cassette, sub-family D, member 2, mRNA [NM_005164] |
| 211 | IL3RA | 6.53 | Homo sapiens interleukin 3 receptor, alpha, mRNA [NM_002183] |
| 212 | PCDH12 | 6.51 | Homo sapiens protocadherin 12, mRNA [NM_016580] |
| 213 | GNAT1 | 6.49 | Homo sapiens guanine nucleotide binding protein (G protein), mRNA [NM_000172] |
| 214 | DHX58 | 6.48 | Homo sapiens DEXH (Asp-Glu-X-His) box polypeptide 58, mRNA [NM_024119] |
| 215 | TAS2R3 | 6.44 | Homo sapiens taste receptor, type 2, member 3, mRNA [NM_016943] |
| 216 | ST8SIA4 | 6.39 | Homo sapiens ST8 alpha-N-acetyl-neuraminide alpha-2,8-sialyltransferase 4, mRNA [NM_005668] |
| 217 | SLC4A11 | 6.39 | Homo sapiens solute carrier family 4, member 11, mRNA [NM_032034] |
| 219 | OR8D2 | 6.32 | Homo sapiens olfactory receptor, family 8, subfamily D, member 2, mRNA [NM_001002918] |
| 220 | C1QL2 | 6.32 | Homo sapiens complement component 1, q subcomponent-like 2, mRNA [NM_182528] |
| 221 | B3GALT5 | 6.30 | Homo sapiens UDP-Gal:betaGlcNAc beta 1,3-galactosyltransferase, 5, mRNA [NM_033173] |
| 222 | CCL26 | 6.27 | Homo sapiens chemokine (C-C motif) ligand 26, mRNA [NM_006072] |
| 223 | PLSCR1 | 6.26 | Homo sapiens phospholipid scramblase 1, mRNA [NM_021105] |
| 224 | CYP4A11 | 6.24 | CYP4A11=fatty acid omega-hydroxylase [S67580] |
| 225 | CAMK1G | 6.22 | Homo sapiens calcium/calmodulin-dependent protein kinase IG, mRNA [NM_020439] |
| 226 | IL4I1 | 6.18 | Homo sapiens interleukin 4 induced 1, transcript variant 2, mRNA [NM_172374] |
| 227 | STAT1 | 6.16 | Homo sapiens signal transducer and activator of transcription 1, mRNA [NM_139266] |
| 228 | ITIH5L | 6.13 | Homo sapiens inter-alpha (globulin) inhibitor H5-like, mRNA [NM_198510] |
| 229 | DTX3L | 6.13 | Homo sapiens deltex 3-like, mRNA [NM_138287] |
| 230 | AGXT2 | 6.10 | Homo sapiens alanine-glyoxylate aminotransferase 2, mRNA [NM_031900] |
| 231 | ACE2 | 6.10 | Homo sapiens angiotensin I converting enzyme 2, mRNA [NM_021804] |
| 232 | ERVWE1 | 6.09 | Homo sapiens endogenous retroviral family W, env(C7), member 1, mRNA [NM_014590] |
| 233 | SNX20 | 6.08 | Homo sapiens sorting nexin 20, transcript variant 2, mRNA [NM_153337] |
| 234 | OR2J2 | 6.05 | Homo sapiens olfactory receptor, family 2, subfamily J, member 2, mRNA [NM_030905] |
| 235 | AIF1 | 6.03 | Homo sapiens allograft inflammatory factor 1, transcript variant 2, mRNA [NM_004847] |
| 236 | NPPB | 6.01 | Homo sapiens natriuretic peptide precursor B, mRNA [NM_002521] |
| 237 | HOXD4 | 5.95 | Homo sapiens homeobox D4, mRNA [NM_014621] |
| 238 | PTPRZ1 | 5.92 | Homo sapiens protein tyrosine phosphatase, receptor-type, Z1, mRNA [NM_002851] |
| 239 | WHSC2 | 5.92 | Homo sapiens Wolf-Hirschhorn syndrome candidate 2, mRNA [NM_005663] |
| 240 | MOBP | 5.92 | Homo sapiens myelin-associated oligodendrocyte basic protein, mRNA [NM_182935] |
| 241 | CDH23 | 5.89 | Homo sapiens cadherin-related 23, transcript variant 1, mRNA [NM_022124] |
| 242 | PIP | 5.87 | Homo sapiens prolactin-induced protein, mRNA [NM_002652] |
| 243 | OR51E2 | 5.86 | Homo sapiens olfactory receptor, family 51, subfamily E, member 2, mRNA [NM_030774] |
| 244 | OAS3 | 5.84 | Homo sapiens 2'-5'-oligoadenylate synthetase 3, mRNA [NM_006187] |
| 245 | OR2S2 | 5.84 | Homo sapiens olfactory receptor, family 2, subfamily S, member 2, mRNA [NM_019897] |
| 246 | RNF183 | 5.84 | Homo sapiens ring finger protein 183, mRNA [NM_145051] |
| 247 | ZCCHC13 | 5.83 | Homo sapiens zinc finger, CCHC domain containing 13, mRNA [NM_203303] |
| 248 | ACSM1 | 5.81 | Homo sapiens acyl-CoA synthetase medium-chain family member 1, mRNA [NM_052956] |
| 249 | BCOR | 5.81 | Homo sapiens BCL6 co-repressor, transcript variant 1, mRNA [NM_017745] |
| 250 | AXIN2 | 5.80 | Homo sapiens conductin mRNA [AF078165] |
| 251 | DIO2 | 5.77 | Homo sapiens deiodinase, iodothyronine, type II, transcript variant 1, mRNA [NM_013989] |
| 252 | GIMAP5 | 5.76 | Homo sapiens GTPase, IMAP family member 5, mRNA [NM_018384] |
| 253 | HLA-DQA1 | 5.75 | Human HLA-DC classII histocompatibility antigens alpha-chain mRN. [X00370] |
| 254 | CELF5 | 5.75 | Homo sapiens CUGBP, Elav-like family member 5, mRNA [NM_021938] |
| 255 | ZBP1 | 5.75 | Homo sapiens Z-DNA binding protein 1, transcript variant 1, mRNA [NM_030776] |
| 256 | TLR3 | 5.73 | Homo sapiens toll-like receptor 3, mRNA [NM_003265] |
| 257 | FIGF | 5.72 | Homo sapiens c-fos induced growth factor, mRNA [NM_004469] |
| 258 | ZNF333 | 5.71 | Homo sapiens zinc finger protein 333 mRNA, [AF372702] |
| 259 | TNFRSF10D | 5.69 | Homo sapiens TNF receptor superfamily, member 10d, mRNA [NM_003840] |
| 260 | LONRF1 | 5.69 | Homo sapiens LON peptidase N-terminal domain and ring finger 1, mRNA [NM_152271] |
| 261 | KLHDC7B | 5.67 | Homo sapiens kelch domain containing 7B, mRNA [NM_138433] |
| 262 | NAT8 | 5.65 | Homo sapiens N-acetyltransferase 8, mRNA [NM_003960] |
| 263 | PKHD1 | 5.65 | Homo sapiens polycystic kidney and hepatic disease 1, mRNA [NM_170724] |
| 264 | CLDN8 | 5.63 | Homo sapiens claudin 8, mRNA [NM_199328] |
| 265 | SPRR2G | 5.63 | Homo sapiens small proline-rich protein 2G mRNA [NM_001014291] |
| 266 | L3MBTL4 | 5.62 | Homo sapiens l(3)mbt-like 4 mRNA [NM_173464] |
| 267 | PLD4 | 5.62 | Homo sapiens phospholipase D family, member 4 mRNA [NM_138790] |
| 268 | BST2 | 5.61 | Homo sapiens bone marrow stromal cell antigen 2, mRNA [NM_004335] |
| 269 | DDX60L | 5.60 | Homo sapiens DEAD (Asp-Glu-Ala-Asp) box polypeptide 60-like, mRNA [NM_001012967] |
| 270 | DNAJA4 | 5.60 | Homo sapiens PRO1472 mRNA [AF116663] |
| 271 | ACTN2 | 5.60 | Homo sapiens actinin, alpha 2, mRNA [NM_001103] |
| 272 | STAT1 | 5.59 | Homo sapiens signal transducer and activator of transcription 1, mRNA [NM_007315] |
| 273 | SOX3 | 5.58 | Homo sapiens SRY (sex determining region Y)-box 3, mRNA [NM_005634] |
| 274 | PLA2G2A | 5.58 | Homo sapiens phospholipase A2, group IIA, transcript variant 1, mRNA [NM_000300] |
| 275 | CLDN14 | 5.57 | Homo sapiens claudin 14, transcript variant 1, mRNA [NM_144492] |
| 276 | SCGN | 5.57 | Homo sapiens secretagogin, EF-hand calcium binding protein, mRNA [NM_006998] |
| 277 | NECAB1 | 5.57 | Homo sapiens N-terminal EF-hand calcium binding protein 1, mRNA [NM_022351] |
| 278 | AQP7 | 5.56 | Homo sapiens aquaporin 7, mRNA [NM_001170] |
| 279 | P2RY12 | 5.54 | Homo sapiens purinergic receptor P2Y, G-protein coupled, 12), mRNA [NM_022788] |
| 280 | RFX4 | 5.51 | Homo sapiens regulatory factor X, 4 transcript variant 3, mRNA [NM_213594] |
| 281 | ZNF510 | 5.51 | Homo sapiens zinc finger protein 510, mRNA [NM_014930] |
| 282 | IFIH1 | 5.45 | Homo sapiens interferon induced with helicase C domain 1, mRNA [NM_022168] |
| 283 | FGF1 | 5.44 | Homo sapiens acid fibroblast growth factor-like protein mRNA [AF211169] |
| 284 | TAS2R40 | 5.43 | Homo sapiens taste receptor, type 2, member 40, mRNA [NM_176882] |
| 285 | XAF1 | 5.43 | Homo sapiens XIAP associated factor 1, transcript variant 1, mRNA [NM_017523] |
| 286 | CFH | 5.41 | Homo sapiens complement factor H, transcript variant 2, mRNA [NM_001014975] |
| 287 | CD34 | 5.40 | Homo sapiens CD34 molecule, transcript variant 2, mRNA [NM_001773] |
| 288 | KRT14 | 5.40 | Homo sapiens keratin 14, mRNA [NM_000526] |
| 289 | MIA2 | 5.38 | Homo sapiens melanoma inhibitory activity 2, mRNA [NM_054024] |
| 290 | XAF1 | 5.38 | Homo sapiens XIAP associated factor 1, transcript variant 1, mRNA [NM_017523] |
| 291 | PRRX1 | 5.35 | Homo sapiens paired related homeobox 1 mRNA [NM_006902] |
| 292 | MAP4K1 | 5.35 | Homo sapiens mitogen-activated protein kinase kinase kinase kinase 1 mRNA [NM_001042600] |
| 293 | NMI | 5.31 | Homo sapiens N-myc (and STAT) interactor, mRNA [NM_004688] |
| 294 | CTXN2 | 5.30 | Homo sapiens cortexin 2, mRNA [NM_001145668] |
| 295 | C1S | 5.30 | Homo sapiens complement component 1, s subcomponent mRNA [NM_001734] |
| 296 | OAS3 | 5.29 | Homo sapiens 2'-5'-oligoadenylate synthetase 3, mRNA [NM_006187] |
| 297 | KRTAP13-3 | 5.28 | Homo sapiens keratin associated protein 13-3, mRNA [NM_181622] |
| 298 | USP26 | 5.27 | Homo sapiens ubiquitin specific peptidase 26, mRNA [NM_031907] |
| 299 | GIMAP2 | 5.26 | Homo sapiens GTPase, IMAP family member 2, mRNA [NM_015660] |
| 300 | ERN2 | 5.25 | Homo sapiens endoplasmic reticulum to nucleus signaling 2, mRNA [NM_033266] |
| 301 | BTNL9 | 5.23 | Homo sapiens butyrophilin-like 9, mRNA [NM_152547] |
| 302 | KLK1 | 5.22 | Homo sapiens kallikrein 1, mRNA [NM_002257] |
| 303 | HCP5 | 5.19 | Homo sapiens HLA complex P5, mRNA [NM_006674] |
| 304 | HDAC9 | 5.18 | Homo sapiens histone deacetylase 9, transcript variant 1, mRNA [NM_058176] |
| 305 | FLT4 | 5.16 | Homo sapiens fms-related tyrosine kinase 4, mRNA [NM_182925] |
| 306 | AIM2 | 5.16 | Homo sapiens absent in melanoma 2, mRNA [NM_004833] |
| 307 | ATP2A3 | 5.16 | Homo sapiens ATPase, Ca++ transporting, ubiquitous, mRNA [NM_174958] |
| 308 | CALN1 | 5.15 | Homo sapiens calneuron 1, transcript variant 1, mRNA [NM_031468] |
| 309 | IFI44 | 5.14 | Homo sapiens interferon-induced protein 44, mRNA [NM_006417] |
| 310 | KRT17 | 5.14 | Homo sapiens keratin 17, mRNA [NM_000422] |
| 311 | C1QTNF9 | 5.14 | Homo sapiens C1q and tumor necrosis factor related protein 9, mRNA [NM_178540] |
| 312 | LRRC55 | 5.14 | Homo sapiens leucine rich repeat containing 55, mRNA [NM_001005210] |
| 313 | IRF7 | 5.13 | Homo sapiens interferon regulatory factor 7, transcript variant d, mRNA [NM_004031] |
| 314 | DCX | 5.13 | Homo sapiens doublecortin, transcript variant 1, mRNA [NM_000555] |
| 315 | NCF1 | 5.12 | Homo sapiens neutrophil cytosolic factor 1, mRNA [NM_000265] |
| 316 | PPP1R16B | 5.12 | Homo sapiens protein phosphatase 1, regulatory subunit 16B mRNA [NM_015568] |
| 317 | BANF2 | 5.12 | Homo sapiens barrier to autointegration factor 2 mRNA [NM_001014977] |
| 318 | WNT1 | 5.11 | Homo sapiens wingless-type MMTV integration site family, member 1, mRNA [NM_005430] |
| 319 | INSRR | 5.11 | Homo sapiens insulin receptor-related receptor, mRNA [NM_014215] |
| 320 | OR4N4 | 5.06 | Homo sapiens olfactory receptor, family 4, subfamily N, member 4, mRNA [NM_001005241] |
| 321 | PPP1R1A | 5.05 | Homo sapiens protein phosphatase 1, regulatory (inhibitor) subunit 1A, mRNA [NM_006741] |
| 322 | ACTG2 | 5.02 | Homo sapiens actin, gamma 2, mRNA [NM_001615] |
| 323 | HS6ST3 | 5.01 | Homo sapiens heparan sulfate 6-O-sulfotransferase 3, mRNA [NM_153456] |
| 324 | ACE | 5.00 | Homo sapiens angiotensin I converting enzyme, transcript variant 1, mRNA [NM_000789] |

***B. Candidate genes down-regulated by 5 folds in miR-145 transfection***

| 1 | CETP | 1821.42 | Homo sapiens cholesteryl ester transfer protein, mRNA [NM_000078] |
| --- | --- | --- | --- |
| 2 | ZPBP2 | 62.14 | Homo sapiens zona pellucida binding protein 2, mRNA [NM_199321] |
| 3 | MAGEB2 | 60.31 | Homo sapiens melanoma antigen family B, 2, mRNA [NM_002364] |
| 4 | GALNT9 | 59.82 | Homo sapiens UDP-N-acetyl-alpha-D-galactosamine:polypeptide N-acetylgalactosaminyltransferase 9, mRNA [NM_021808] |
| 5 | POF1B | 56.25 | Homo sapiens premature ovarian failure, 1B, mRNA [NM_024921] |
| 6 | TTLL6 | 49.83 | Homo sapiens tubulin tyrosine ligase-like family, member 6 mRNA [NM_173623] |
| 7 | PHC2 | 49.65 | Homo sapiens polyhomeotic homolog 2, transcript variant 1, mRNA [NM_198040] |
| 8 | FERMT1 | 48.74 | Homo sapiens fermitin family homolog 1, mRNA [NM_017671] |
| 9 | KDM6B | 41.77 | Homo sapiens lysine (K)-specific demethylase 6B, mRNA [NM_001080424] |
| 10 | MCF2L | 38.26 | Homo sapiens MCF.2 cell line derived transforming sequence-like mRNA [NM_024979] |
| 11 | LPPR5 | 35.06 | Homo sapiens lipid phosphate phosphatase-related protein type 5 mRNA [NM_001010861] |
| 12 | ABO | 33.75 | Homo sapiens ABO blood group (transferase A), mRNA [NM_020469] |
| 13 | CRTAM | 33.17 | Homo sapiens cytotoxic and regulatory T cell molecule, mRNA [NM_019604] |
| 14 | KCNMA1 | 32.77 | Homo sapiens K large conductance Ca-activated channel M, alpha 1, mRNA [NM_002247] |
| 15 | GABRG1 | 32.32 | Homo sapiens gamma-aminobutyric acid A receptor, gamma 1, mRNA [NM_173536] |
| 16 | ITGAX | 32.20 | Homo sapiens integrin, alpha X, mRNA [NM_000887] |
| 17 | PNLIP | 30.25 | Homo sapiens pancreatic lipase, mRNA [NM_000936] |
| 18 | NGB | 29.62 | Homo sapiens neuroglobin, mRNA [NM_021257] |
| 19 | FASLG | 28.30 | Homo sapiens Fas ligand, mRNA [NM_000639] |
| 20 | NLRP7 | 27.08 | Homo sapiens NLR family, pyrin domain containing 7 mRNA [NM_139176] |
| 21 | IFT57 | 26.36 | Homo sapiens intraflagellar transport 57 homolog, mRNA [NM_018010] |
| 22 | MYRIP | 26.23 | Homo sapiens myosin VIIA and Rab interacting protein, mRNA [NM_015460] |
| 23 | ADAMTS14 | 25.89 | Homo sapiens ADAM metallopeptidase with thrombospondin type 1, mRNA [NM_139155] |
| 24 | BEST1 | 25.36 | Homo sapiens bestrophin 1, transcript variant 1, mRNA [NM_004183] |
| 25 | TMEM71 | 25.33 | Homo sapiens transmembrane protein 71 mRNA [NM_144649] |
| 26 | EN1 | 24.70 | Homo sapiens engrailed homeobox 1, mRNA [NM_001426] |
| 27 | MMRN1 | 24.44 | Homo sapiens multimerin 1, mRNA [NM_007351] |
| 28 | XCL2 | 24.29 | Homo sapiens chemokine (C motif) ligand 2, mRNA [NM_003175] |
| 29 | DEFB119 | 23.76 | Homo sapiens defensin, beta 119, transcript variant 2, mRNA [NM_173460] |
| 30 | SCN7A | 23.57 | Sodium channel, voltage-gated, type VII, alpha [ENST00000409855] |
| 31 | B4GALNT3 | 23.51 | Homo sapiens beta-1,4-N-acetyl-galactosaminyl transferase 3, mRNA [NM_173593] |
| 32 | NLN | 23.48 | Homo sapiens neurolysin (metallopeptidase M3 family), mRNA [BC004985] |
| 33 | LY6H | 23.18 | Homo sapiens lymphocyte antigen 6 complex, locus H, mRNA [NM_002347] |
| 34 | LRFN5 | 22.77 | Homo sapiens leucine rich repeat and fibronectin type III domain containing 5, mRNA [NM_152447] |
| 35 | RGNEF | 22.63 | Homo sapiens Rho-guanine nucleotide exchange factor, mRNA [NM_001080479] |
| 36 | GBX1 | 22.07 | Homo sapiens gastrulation brain homeobox 1, mRNA [NM_001098834] |
| 37 | FAM149A | 21.81 | Homo sapiens family with sequence similarity 149, member A, mRNA [NM_015398] |
| 38 | ZSCAN4 | 21.16 | Homo sapiens zinc finger and SCAN domain containing 4, mRNA [NM_152677] |
| 39 | ADAMTS8 | 21.11 | Homo sapiens ADAM metallopeptidase with thrombospondin type 1, 8, mRNA [NM_007037] |
| 40 | GIP | 21.08 | Homo sapiens gastric inhibitory polypeptide, mRNA [NM_004123] |
| 41 | BMX | 20.15 | Homo sapiens BMX non-receptor tyrosine kinase mRNA [NM_001721] |
| 42 | SOX5 | 19.76 | Homo sapiens SRY (sex determining region Y)-box 5 mRNA [NM_152989] |
| 43 | SEMA3E | 18.91 | Homo sapiens sema domain, Ig short basic domain, secreted, 3E mRNA [NM_012431] |
| 44 | HAPLN4 | 18.44 | Homo sapiens hyaluronan and proteoglycan link protein 4, mRNA [NM_023002] |
| 45 | MAGEC2 | 18.41 | Homo sapiens melanoma antigen family C, 2, mRNA [NM_016249] |
| 46 | PCDHGC5 | 18.41 | Homo sapiens protocadherin gamma subfamily C, 5, mRNA [NM_032407] |
| 47 | CNR2 | 18.35 | Cannabinoid receptor 2 [ENST00000374472] |
| 48 | NPHS2 | 18.32 | Homo sapiens nephrosis 2, idiopathic, steroid-resistant (podocin), mRNA [NM_014625] |
| 49 | OR51B4 | 18.14 | Homo sapiens olfactory receptor, family 51, subfamily B, member 4, mRNA [NM_033179] |
| 50 | SLAMF1 | 18.02 | Homo sapiens signaling lymphocytic activation molecule family member 1, mRNA [NM_003037] |
| 51 | EDDM3B | 18.00 | Homo sapiens epididymal protein 3B, mRNA [NM_022360] |
| 52 | NCAM1 | 17.68 | Homo sapiens neural cell adhesion molecule 1, mRNA [NM_001076682] |
| 53 | IP6K3 | 17.38 | Homo sapiens inositol hexakisphosphate kinase 3 mRNA [NM_054111] |
| 54 | ADAMTSL3 | 17.34 | Homo sapiens ADAMTS-like 3, mRNA [NM_207517] |
| 55 | PCDHB12 | 17.14 | Homo sapiens protocadherin beta 12, mRNA [NM_018932] |
| 56 | C2 | 16.82 | Homo sapiens complement component 2, mRNA [BC029781] |
| 57 | COL15A1 | 16.60 | Homo sapiens collagen, type XV, alpha 1, mRNA [NM_001855] |
| 58 | CDH6 | 16.57 | Cadherin 6, type 2, K-cadherin [ENST00000265071] |
| 59 | CD37 | 16.44 | Homo sapiens CD37 molecule, transcript variant 1, mRNA [NM_001774] |
| 60 | FAM107A | 16.28 | Homo sapiens family with sequence similarity 107, member A mRNA [NM_007177] |
| 61 | SCNN1G | 16.21 | H.sapiens mRNA for gamma subunit of epithelial amiloride-sensitive Na channel [X87160] |
| 62 | NLRP13 | 16.15 | Homo sapiens NLR family, pyrin domain containing 13, mRNA [NM_176810] |
| 63 | SH2D4B | 15.94 | Homo sapiens SH2 domain containing 4B, transcript variant 1, mRNA [NM_207372] |
| 64 | CD8B | 15.73 | Homo sapiens CD8b molecule, transcript variant 1, mRNA [NM_172099] |
| 65 | TIGIT | 15.67 | Homo sapiens T cell immunoreceptor with Ig and ITIM domains, mRNA [NM_173799] |
| 66 | THAP6 | 15.58 | Homo sapiens THAP domain containing 6, mRNA [NM_144721] |
| 67 | APCS | 15.54 | Homo sapiens amyloid P component, mRNA [NM_001639] |
| 68 | OR2T8 | 15.30 | Homo sapiens olfactory receptor, family 2, subfamily T, member 8, mRNA [NM_001005522] |
| 69 | FERD3L | 15.21 | Homo sapiens Fer3-like, mRNA [NM_152898] |
| 70 | TRHDE | 14.80 | Homo sapiens thyrotropin-releasing hormone degrading enzyme, mRNA [NM_013381] |
| 71 | RFX4 | 14.79 | Homo sapiens regulatory factor X, 4 mRNA [NM_002920] |
| 72 | UNC45B | 14.49 | Homo sapiens unc-45 homolog B, transcript variant 1, mRNA [NM_173167] |
| 73 | CELA2A | 14.41 | Homo sapiens chymotrypsin-like elastase family, member 2A, mRNA [NM_033440] |
| 74 | TAS2R42 | 14.41 | Homo sapiens taste receptor, type 2, member 42, mRNA [NM_181429] |
| 75 | PFKFB1 | 14.34 | Homo sapiens 6-phosphofructo-2-kinase/fructose-2,6-biphosphatase 1, mRNA [NM_002625] |
| 76 | LYZ | 14.32 | Homo sapiens lysozyme, mRNA [NM_000239] |
| 77 | VSIG4 | 14.08 | Homo sapiens V-set and Ig domain containing 4, mRNA [NM_007268] |
| 78 | LCN6 | 14.06 | Homo sapiens lipocalin 6, mRNA [NM_198946] |
| 79 | LILRA5 | 14.01 | Homo sapiens leukocyte Ig-like receptor, subfamily A, member 5 mRNA [NM_181879] |
| 80 | FGF9 | 13.99 | Homo sapiens fibroblast growth factor 9, mRNA [NM_002010] |
| 81 | PDYN | 13.98 | Homo sapiens prodynorphin, mRNA [NM_024411] |
| 82 | MS4A5 | 13.96 | Homo sapiens membrane-spanning 4-domains, subfamily A, 5, mRNA [NM_023945] |
| 83 | SLA2 | 13.90 | Homo sapiens Src-like-adaptor 2, transcript variant 1, mRNA [NM_032214] |
| 84 | HLA-DPB1 | 13.88 | Homo sapiens major histocompatibility complex, class II, DP beta 1, mRNA [NM_002121] |
| 85 | MYOZ2 | 13.86 | Homo sapiens myozenin 2, mRNA [NM_016599] |
| 86 | ITGA9 | 13.84 | Homo sapiens integrin, alpha 9, mRNA [NM_002207] |
| 87 | CADM4 | 13.78 | Homo sapiens cell adhesion molecule 4, mRNA [NM_145296] |
| 88 | SCN10A | 13.70 | Homo sapiens sodium channel, voltage-gated, type X, alpha subunit, mRNA [NM_006514] |
| 89 | RECQL | 13.37 | Homo sapiens RecQ protein-like, mRNA [NM_002907] |
| 90 | CELF4 | 13.37 | Homo sapiens LYST-interacting protein LIP9 mRNA [AF141345] |
| 91 | ARG1 | 13.27 | Homo sapiens arginase, mRNA [NM_000045] |
| 92 | SLC6A20 | 13.25 | Homo sapiens solute carrier family 6, member 20 mRNA [NM_020208] |
| 93 | PCSK6 | 13.19 | Homo sapiens proprotein convertase subtilisin/kexin type 6 mRNA [NM_138322] |
| 94 | DLC1 | 13.05 | Homo sapiens deleted in liver cancer 1 mRNA [NM_024767] |
| 95 | EPHA7 | 13.01 | Homo sapiens EPH receptor A7, mRNA [BC027940] |
| 96 | ATOH8 | 12.97 | Homo sapiens atonal homolog 8, mRNA [NM_032827] |
| 97 | SLC36A2 | 12.97 | Homo sapiens solute carrier family 36, member 2, mRNA [NM_181776] |
| 98 | MYST4 | 12.83 | Homo sapiens MYST histone acetyltransferase 4, mRNA [NM_012330] |
| 99 | HYDIN | 12.80 | Homo sapiens hydrocephalus inducing homolog, mRNA [NM_032821] |
| 100 | ARHGEF17 | 12.75 | Homo sapiens Rho guanine nucleotide exchange factor 17, mRNA [NM_014786] |
| 101 | ANGPT4 | 12.66 | Homo sapiens angiopoietin 4, mRNA [NM_015985] |
| 102 | GFRA1 | 12.57 | Homo sapiens GDNF family receptor alpha 1, mRNA [NM_145793] |
| 103 | PPEF2 | 12.53 | Homo sapiens protein phosphatase, EF-hand Ca binding domain 2, mRNA [NM_006239] |
| 104 | ZNF711 | 12.44 | Homo sapiens zinc finger protein 711, mRNA [NM_021998] |
| 105 | PDZRN4 | 12.25 | Homo sapiens PDZ domain containing ring finger 4 mRNA [NM_013377] |
| 106 | IL1RAPL2 | 12.20 | Homo sapiens interleukin 1 receptor accessory protein-like 2, mRNA [NM_017416] |
| 107 | PDGFRA | 12.17 | Homo sapiens platelet-derived growth factor receptor, alpha, mRNA [NM_006206] |
| 108 | TAC1 | 12.03 | Homo sapiens tachykinin, precursor 1, transcript variant beta, mRNA [NM_003182] |
| 109 | GPR35 | 11.94 | Homo sapiens G protein-coupled receptor 35, mRNA [NM_005301] |
| 110 | ZNF417 | 11.90 | Homo sapiens zinc finger protein 417, mRNA [NM_152475] |
| 111 | TBX15 | 11.88 | Homo sapiens T-box 15, mRNA [NM_152380] |
| 112 | PDE6G | 11.87 | Homo sapiens phosphodiesterase 6G, cGMP-specific, rod, gamma mRNA [NM_002602] |
| 113 | SPINLW1 | 11.86 | Homo sapiens serine peptidase inhibitor-like, with Kunitz and WAP domains 1 mRNA [NM_181502] |
| 114 | RASSF6 | 11.79 | Homo sapiens Ras association domain family member 6 mRNA [NM_201431] |
| 115 | KCNN3 | 11.76 | Homo sapiens K intermediate/small conductance Ca-activated channel, subfamily N, member 3 mRNA [NM_002249] |
| 116 | PRPH2 | 11.66 | Homo sapiens peripherin 2 mRNA [NM_000322] |
| 117 | UGT2B4 | 11.54 | Homo sapiens UDP glucuronosyltransferase 2 family, polypeptide B4 mRNA [NM_021139] |
| 118 | LCN12 | 11.29 | Homo sapiens lipocalin 12 mRNA [NM_178536] |
| 119 | GSTT1 | 11.24 | Homo sapiens glutathione S-transferase theta 1, mRNA [NM_000853] |
| 120 | REG4 | 11.21 | Homo sapiens regenerating islet-derived family, member 4 mRNA [NM_032044] |
| 121 | PATE1 | 11.08 | Homo sapiens prostate and testis expressed 1, mRNA [NM_138294] |
| 122 | CPXCR1 | 10.92 | Homo sapiens CPX chromosome region, candidate 1, mRNA [NM_033048] |
| 123 | FCGR2B | 10.92 | Homo sapiens Fc fragment of IgG, low affinity IIb, receptor (CD32), mRNA [NM_004001] |
| 124 | ST6GAL1 | 10.91 | Homo sapiens ST6 beta-galactosamide alpha-2,6-sialyltranferase 1 mRNA [NM_173216] |
| 125 | KCNV1 | 10.84 | Homo sapiens potassium channel, subfamily V, member 1, mRNA [NM_014379] |
| 126 | KLHL30 | 10.82 | Homo sapiens kelch-like 30, mRNA [NM_198582] |
| 127 | ELF5 | 10.81 | Homo sapiens E74-like factor 5, mRNA [NM_198381] |
| 128 | SGCD | 10.75 | Homo sapiens sarcoglycan, delta mRNA [NM_172244] |
| 129 | HIGD1B | 10.74 | Homo sapiens HIG1 hypoxia inducible domain family, member 1B, mRNA [NM_016438] |
| 130 | HIST2H2BE | 10.45 | Homo sapiens histone cluster 2, H2be, mRNA [NM_003528] |
| 131 | FCRLA | 10.42 | Homo sapiens Fc receptor-like A (FCRLA), mRNA [NM_032738] |
| 132 | SPON1 | 10.29 | Homo sapiens spondin 1, mRNA [NM_006108] |
| 133 | SH2D5 | 10.15 | Homo sapiens SH2 domain containing 5, mRNA [NM_001103161] |
| 134 | KCNMB2 | 9.96 | Homo sapiens K large conductance Ca-activated channel, subfamily M, beta 2, mRNA [NM_181361] |
| 135 | LOXL4 | 9.95 | Homo sapiens lysyl oxidase-like 4, mRNA [NM_032211] |
| 136 | ERBB2 | 9.86 | Homo sapiens v-erb-b2 erythroblastic leukemia viral oncogene homolog 2, mRNA [NM_001005862] |
| 137 | PRO2949 | 9.79 | Homo sapiens PRO2949 mRNA [AF119907] |
| 138 | SAP130 | 9.77 | Homo sapiens Sin3A-associated protein, mRNA [NM_001145928] |
| 139 | FGF14 | 9.61 | Homo sapiens fibroblast growth factor 14, mRNA [NM_175929] |
| 140 | PKHD1L1 | 9.58 | Homo sapiens polycystic kidney and hepatic disease 1-like 1, mRNA [NM_177531] |
| 141 | TTC29 | 9.55 | Homo sapiens tetratricopeptide repeat domain 29, mRNA [NM_031956] |
| 142 | FAM71D | 9.46 | Homo sapiens family with sequence similarity 71, member D, mRNA [NM_173526] |
| 143 | NPAS4 | 9.36 | Homo sapiens neuronal PAS domain protein 4, mRNA [NM_178864] |
| 144 | NAP1L2 | 9.26 | Homo sapiens nucleosome assembly protein 1-like 2, mRNA [NM_021963] |
| 145 | FXYD1 | 9.25 | Homo sapiens FXYD domain containing ion transport regulator 1 mRNA [NM_005031] |
| 146 | PSCA | 9.21 | Homo sapiens prostate stem cell antigen mRNA [NM_005672] |
| 147 | ALPI | 8.96 | Homo sapiens alkaline phosphatase, mRNA [NM_001631] |
| 148 | TTC28 | 8.93 | Homo sapiens tetratricopeptide repeat domain 28, mRNA [NM_001145418] |
| 149 | RAX | 8.92 | Homo sapiens retina and anterior neural fold homeobox, mRNA [NM_013435] |
| 150 | CALML6 | 8.92 | Homo sapiens calmodulin-like 6, mRNA [NM_138705] |
| 151 | MOV10L1 | 8.91 | Homo sapiens Mov10l1, Moloney leukemia virus 10-like 1, homolog, mRNA [NM_018995] |
| 152 | SYN1 | 8.87 | Homo sapiens synapsin I, transcript variant Ia, mRNA [NM_006950] |
| 153 | GLRA4 | 8.81 | Homo sapiens glycine receptor, alpha 4, transcript variant 1, mRNA [NM_001024452] |
| 154 | ASB4 | 8.75 | Ankyrin repeat and SOCS box-containing 4 [ENST00000325885] |
| 155 | GPR65 | 8.73 | Homo sapiens G protein-coupled receptor 65, mRNA [NM_003608] |
| 156 | SST | 8.55 | Homo sapiens somatostatin, mRNA [NM_001048] |
| 157 | BPIL1 | 8.45 | Homo sapiens bactericidal/permeability-increasing protein-like 1, mRNA [NM_025227] |
| 158 | SOST | 8.42 | Homo sapiens sclerosteosis, mRNA [NM_025237] |
| 159 | TBR1 | 8.40 | Homo sapiens T-box, brain, 1, mRNA [NM_006593] |
| 160 | HLA-DQA1 | 8.40 | Homo sapiens major histocompatibility complex, class II, DQ alpha 1, mRNA [NM_002122] |
| 161 | KIR3DL2 | 8.17 | Homo sapiens killer cell Ig-like receptor, three domains, long cytoplasmic tail, 2, mRNA [NM_006737] |
| 162 | FAM90A1 | 8.14 | Homo sapiens family with sequence similarity 90, member A1, mRNA [NM_018088] |
| 163 | FLOT2 | 8.08 | Homo sapiens flotillin 2, mRNA [NM_004475] |
| 164 | LEUTX | 8.05 | Homo sapiens leucine twenty homeobox, mRNA [NM_001143832] |
| 165 | DRD1 | 8.01 | Homo sapiens dopamine receptor D1, mRNA [NM_000794] |
| 166 | HTR3A | 7.98 | Homo sapiens 5-hydroxytryptamine (serotonin) receptor 3A, mRNA [NM_213621] |
| 167 | WBP2NL | 7.97 | Homo sapiens WBP2 N-terminal like, mRNA [NM_152613] |
| 168 | MC5R | 7.88 | Homo sapiens melanocortin 5 receptor, mRNA [NM_005913] |
| 169 | DSEL | 7.85 | Homo sapiens dermatan sulfate epimerase-like, mRNA [NM_032160] |
| 170 | HHATL | 7.81 | Homo sapiens hedgehog acyltransferase-like, mRNA [NM_020707] |
| 171 | FAM123C | 7.79 | Homo sapiens family with sequence similarity 123C, mRNA [NM_152698] |
| 172 | PSIP1 | 7.75 | Homo sapiens PC4 and SFRS1 interacting protein 1, mRNA [BC033817] |
| 173 | MT1JP | 7.74 | Homo sapiens MTB mRNA [AF348994] |
| 174 | APLF | 7.71 | Homo sapiens chromosome 2 open reading frame 13, mRNA [BC030711] |
| 175 | BIRC8 | 7.67 | Homo sapiens baculoviral IAP repeat-containing 8, mRNA [NM_033341] |
| 176 | ACSM2B | 7.67 | Homo sapiens acyl-CoA synthetase medium-chain family member 2B mRNA [NM_182617] |
| 177 | ADPRH | 7.65 | Homo sapiens ADP-ribosylarginine hydrolase, mRNA [NM_001125] |
| 178 | IGFALS | 7.62 | Homo sapiens insulin-like growth factor binding protein, acid labile subunit, mRNA [NM_004970] |
| 179 | CKMT2 | 7.59 | Homo sapiens creatine kinase, mitochondrial 2 mRNA [NM_001825] |
| 180 | POTED | 7.58 | Homo sapiens POTE ankyrin domain family, member D mRNA [NM_174981] |
| 181 | KRT2 | 7.55 | Homo sapiens keratin 2, mRNA [NM_000423] |
| 182 | HTR6 | 7.46 | Homo sapiens 5-hydroxytryptamine (serotonin) receptor 6, mRNA [NM_000871] |
| 183 | DNAH5 | 7.46 | Homo sapiens dynein, axonemal, heavy chain 5, mRNA [NM_001369] |
| 184 | UNC13A | 7.42 | Homo sapiens unc-13 homolog A, mRNA [NM_001080421] |
| 185 | WFDC10A | 7.37 | Homo sapiens WAP four-disulfide core domain 10A, mRNA [NM_080753] |
| 186 | FBXW2 | 7.36 | Homo sapiens F-box and WD repeat domain containing 2, mRNA [NM_012164] |
| 187 | FHAD1 | 7.35 | Homo sapiens forkhead-associated phosphopeptide binding domain 1, mRNA [NM_052929] |
| 188 | EFNA2 | 7.33 | Homo sapiens ephrin-A2, mRNA [NM_001405] |
| 189 | BFSP2 | 7.29 | Homo sapiens beaded filament structural protein 2, phakinin, mRNA [NM_003571] |
| 190 | NFATC2 | 7.22 | Homo sapiens nuclear factor of activated T-cells, calcineurin-dependent 2, mRNA [NM_173091] |
| 191 | AR | 7.14 | Homo sapiens androgen receptor, transcript variant 1, mRNA [NM_000044] |
| 192 | RHOJ | 7.13 | Homo sapiens ras homolog gene family, member J, mRNA [NM_020663] |
| 193 | PLEKHG4B | 7.02 | Homo sapiens pleckstrin homology domain containing, family G member 4B, mRNA [NM_052909] |
| 194 | CCL7 | 6.97 | Homo sapiens chemokine (C-C motif) ligand 7, mRNA [NM_006273] |
| 196 | LRRTM4 | 6.93 | Homo sapiens leucine rich repeat transmembrane neuronal 4, mRNA [NM_001134745] |
| 197 | ARR3 | 6.92 | Homo sapiens arrestin 3, retinal, mRNA [NM_004312] |
| 198 | SEMG1 | 6.87 | Homo sapiens semenogelin I, mRNA [NM_003007] |
| 199 | RGR | 6.86 | Homo sapiens retinal G protein coupled receptor, mRNA [NM_002921] |
| 200 | MLLT6 | 6.83 | Homo sapiens myeloid/lymphoid or mixed-lineage leukemia; translocated to, 6, mRNA [NM_005937] |
| 201 | DMRT3 | 6.82 | Homo sapiens doublesex and mab-3 related transcription factor 3, mRNA [NM_021240] |
| 202 | ADAMTS6 | 6.78 | Homo sapiens ADAM metallopeptidase with thrombospondin type 1, 6, mRNA [NM_197941] |
| 203 | SERPINE3 | 6.77 | Homo sapiens serpin peptidase inhibitor, clade E member 3, mRNA [NM_001101320] |
| 204 | LPIN2 | 6.75 | Homo sapiens lipin 2, mRNA [NM_014646] |
| 205 | PRO1483 | 6.71 | Homo sapiens PRO1483 mRNA [AF116635] |
| 206 | SYT11 | 6.70 | Homo sapiens synaptotagmin XI, mRNA [NM_152280] |
| 207 | CYP4F11 | 6.69 | Homo sapiens cytochrome P450, family 4, subfamily F, polypeptide 11 mRNA [NM_021187] |
| 208 | KCNAB3 | 6.66 | Homo sapiens K voltage-gated channel, shaker-related subfamily, beta 3 mRNA [NM_004732] |
| 209 | EFHC2 | 6.64 | Homo sapiens EF-hand domain (C-terminal) containing 2, mRNA [NM_025184] |
| 210 | RYR1 | 6.62 | Homo sapiens ryanodine receptor 1, transcript variant 2, mRNA [NM_001042723] |
| 211 | ZSCAN10 | 6.60 | Homo sapiens zinc finger and SCAN domain containing 10, mRNA [NM_032805] |
| 212 | MYOZ1 | 6.55 | Homo sapiens myozenin 1, mRNA [NM_021245] |
| 213 | EML6 | 6.49 | Homo sapiens echinoderm microtubule associated protein like 6, mRNA [NM_001039753] |
| 214 | TTC18 | 6.48 | Homo sapiens tetratricopeptide repeat domain 18, mRNA [NM_145170] |
| 215 | HIST1H1A | 6.44 | Homo sapiens histone cluster 1, H1a, mRNA [NM_005325] |
| 216 | MUC5B | 6.42 | Homo sapiens mucin 5B, oligomeric mucus/gel-forming, mRNA [NM_002458] |
| 217 | PNMA2 | 6.38 | Homo sapiens paraneoplastic antigen MA2, mRNA [NM_007257] |
| 218 | HOXC4 | 6.34 | Homo sapiens homeobox C4, transcript variant 1, mRNA [NM_014620] |
| 219 | FLG | 6.31 | Homo sapiens filaggrin, mRNA [NM_002016] |
| 220 | ANXA13 | 6.24 | Homo sapiens annexin A13, transcript variant 2, mRNA [NM_001003954] |
| 221 | NR5A2 | 6.24 | Homo sapiens nuclear receptor subfamily 5, group A, member 2 mRNA [NM_205860] |
| 222 | NUDT10 | 6.22 | Homo sapiens nudix (nucleoside diphosphate linked moiety X)-type motif 10 mRNA [NM_153183] |
| 223 | FLRT1 | 6.19 | Homo sapiens fibronectin leucine rich transmembrane protein 1, mRNA [NM_013280] |
| 224 | NLRP14 | 6.15 | Homo sapiens NLR family, pyrin domain containing 14, mRNA [NM_176822] |
| 225 | TSPAN32 | 6.13 | Homo sapiens tetraspanin 32, mRNA [NM_139022] |
| 226 | MYO1G | 6.09 | Homo sapiens myosin IG, mRNA [NM_033054] |
| 227 | CD300A | 6.02 | Homo sapiens CD300a molecule, mRNA [NM_007261] |
| 228 | COG6 | 6.02 | Homo sapiens component of oligomeric golgi complex 6 mRNA [NM_001145079] |
| 229 | LRCH1 | 6.01 | Homo sapiens leucine-rich repeats and calponin homology domain containing 1 mRNA [NM_015116] |
| 230 | EPB41L4B | 5.99 | Homo sapiens erythrocyte membrane protein band 4.1 like 4B mRNA [NM_019114] |
| 231 | PKHD1 | 5.99 | Homo sapiens polycystic kidney and hepatic disease 1 mRNA [NM_138694] |
| 232 | SFTPA1 | 5.96 | Homo sapiens surfactant protein A1 mRNA [NM_005411] |
| 233 | TBC1D21 | 5.91 | Homo sapiens TBC1 domain family, member 21, mRNA [NM_153356] |
| 234 | CXCL6 | 5.91 | Homo sapiens chemokine (C-X-C motif) ligand 6, mRNA [NM_002993] |
| 235 | KRTAP8-1 | 5.91 | Homo sapiens keratin associated protein 8-1 mRNA [NM_175857] |
| 236 | ATCAY | 5.90 | Homo sapiens ataxia, cerebellar, mRNA [NM_033064] |
| 237 | CEBPA | 5.84 | Homo sapiens CCAAT/enhancer binding protein, alpha, mRNA [NM_004364] |
| 238 | PBOV1 | 5.83 | Homo sapiens prostate and breast cancer overexpressed 1, mRNA [NM_021635] |
| 239 | GCG | 5.81 | Homo sapiens glucagon, mRNA [NM_002054] |
| 240 | NAF1 | 5.79 | Homo sapiens nuclear assembly factor 1 homolog, mRNA [NM_138386] |
| 241 | TMEM20 | 5.74 | Homo sapiens transmembrane protein 20, mRNA [NM_153226] |
| 242 | GOT1L1 | 5.72 | Homo sapiens glutamic-oxaloacetic transaminase 1-like 1, mRNA [NM_152413] |
| 243 | MLEC | 5.71 | Homo sapiens malectin, mRNA [NM_014730] |
| 244 | SLC35F1 | 5.69 | Homo sapiens solute carrier family 35, member F1, mRNA [NM_001029858] |
| 245 | CACNG7 | 5.62 | Homo sapiens calcium channel, voltage-dependent, gamma subunit 7, mRNA [NM_031896] |
| 246 | AHNAK | 5.61 | Homo sapiens AHNAK nucleoprotein, transcript variant 1, mRNA [NM_001620] |
| 247 | TAS2R8 | 5.49 | Homo sapiens taste receptor, type 2, member 8, mRNA [NM_023918] |
| 248 | PHOSPHO1 | 5.49 | Homo sapiens phosphatase, orphan 1, transcript variant 2, mRNA [NM_178500] |
| 249 | GPR78 | 5.49 | Homo sapiens G protein-coupled receptor 78, mRNA [NM_080819] |
| 250 | MRGPRX4 | 5.47 | Homo sapiens MAS-related GPR, member X4, mRNA [NM_054032] |
| 251 | GRAMD2 | 5.42 | Homo sapiens GRAM domain containing 2, mRNA [NM_001012642] |
| 252 | PIAS3 | 5.42 | Homo sapiens protein inhibitor of activated STAT, 3, mRNA [NM_006099] |
| 253 | PDLIM2 | 5.41 | Homo sapiens PDZ and LIM domain 2, transcript variant 1, mRNA [NM_176871] |
| 254 | HIST1H4B | 5.39 | Homo sapiens histone cluster 1, H4b, mRNA [NM_003544] |
| 255 | KATNAL2 | 5.36 | Homo sapiens katanin p60 subunit A-like 2, mRNA [NM_031303] |
| 256 | F9 | 5.35 | Homo sapiens coagulation factor IX, mRNA [NM_000133] |
| 257 | MYBPH | 5.34 | Homo sapiens myosin binding protein H, mRNA [NM_004997] |
| 258 | ATP10A | 5.33 | Homo sapiens ATP10C mRNA [AB051358] |
| 259 | TDRD5 | 5.32 | Homo sapiens tudor domain containing 5, mRNA [NM_173533] |
| 260 | PPBP | 5.28 | Homo sapiens pro-platelet basic protein ligand 7), mRNA [NM_002704] |
| 261 | DNAH9 | 5.28 | Homo sapiens dynein, axonemal, heavy chain 9, mRNA [NM_001372] |
| 262 | ANKRD31 | 5.27 | Homo sapiens ankyrin repeat domain 31, mRNA [NM_001164443] |
| 263 | ART1 | 5.25 | Homo sapiens ADP-ribosyltransferase 1, mRNA [NM_004314] |
| 264 | HIST1H4D | 5.25 | Homo sapiens histone cluster 1, H4d, mRNA [NM_003539] |
| 265 | TNN | 5.22 | Homo sapiens tenascin N, mRNA [NM_022093] |
| 266 | GUCY2C | 5.18 | Homo sapiens guanylate cyclase 2C, mRNA [NM_004963] |
| 267 | SLC12A1 | 5.17 | Homo sapiens solute carrier family 12, member 1 , mRNA [NM_000338] |
| 268 | FOXJ1 | 5.16 | Homo sapiens forkhead box J1, mRNA [NM_001454] |
| 269 | SH2D6 | 5.15 | Homo sapiens SH2 domain containing 6, mRNA [BC110073] |
| 270 | PPP1R14D | 5.14 | Homo sapiens protein phosphatase 1, regulatory subunit 14D mRNA [NM_017726] |
| 271 | TNXB | 5.12 | Homo sapiens tenascin XB mRNA [NM_019105] |
| 272 | PDK3 | 5.12 | Homo sapiens pyruvate dehydrogenase kinase, isozyme 3 mRNA [NM_001142386] |
| 273 | ODZ2 | 5.08 | Homo sapiens odz, odd Oz/ten-m homolog 2, mRNA [NM_001122679] |
| 274 | BMX | 5.08 | Homo sapiens BMX non-receptor tyrosine kinase, transcript variant 2, mRNA [NM_001721] |
| 275 | ZIC5 | 5.08 | Homo sapiens Zic family member 5, mRNA [NM_033132] |
| 276 | TCTE3 | 5.07 | Homo sapiens t-complex-associated-testis-expressed 3, mRNA [NM_174910] |
| 277 | CACNA2D4 | 5.04 | Homo sapiens calcium channel, voltage-dependent, alpha 2/delta subunit 4, mRNA [NM_172364] |
