# Supplementary material for: MicroRNA-145 Regulates Human Corneal Epithelial Differentiation
Source: PLoS One. 2011 Jun 20;6(6):e21249. doi: 10.1371/journal.pone.0021249 (PMC3119052; doi:10.1371/journal.pone.0021249)
Supplement: Table S4 — Significant Gene Ontology (GO) terms enriched in differential expressed gene list of miR-145- versus scrambled sequence-transfected cells (fold change ≥5). (DOC) [file pone.0021249.s007.doc]

**Table S4. Significant Gene Ontology (GO) terms enriched in differential expressed gene list of miR-145- versus scrambled sequence-transfected cells (fold change ≥ 5)**

| Summarized GO terms | **GO Terms** | **GO Accession** | P-values | **-log(P-value)** |
| --- | --- | --- | --- | --- |
| Immune response process and regulation | Immune response | GO:0006955 | 2.1E-08 | 7.670 |
|  | Immune system process | GO:0002376 | 1.7E-06 | 5.779 |
|  | Regulation of immune system process | GO:0002682 | 6.5E-04 | 3.187 |
|  | Positive regulation of immune system process | GO:0002684 | 9.2E-04 | 3.036 |
|  | Regulation of immune response | GO:0050776 | 1.4E-03 | 2.849 |
| Inflammatory response | Inflammatory response | GO:0006954 | 1.7E-05 | 4.766 |
| Defence response | Defence response | GO:0006952; GO:0002217; GO:0042829 | 1.3E-06 | 5.900 |
| Response to stimulus related GO terms | Response to stimulus | GO:0050896; GO:0051869 | 1.7E-04 | 3.774 |
|  | Positive regulation of response to stimulus | GO:0048584 | 6.4E-04 | 3.192 |
|  | Response to external stimulus | GO:0009605 | 6.6E-04 | 3.182 |
| Regulation of apoptosis and cell death | Regulation of apoptosis | GO:0042981 | 2.1E-03 | 2.673 |
|  | Regulation of programmed cell death | GO:0043067; GO:0043070 | 2.4E-03 | 2.617 |
| Regulation of timing of cell differentiation | Regulation of timing of cell differentiation | GO:0048505 | 2.2E-03 | 2.652 |
| Regulation of development, heterochronic | Regulation of development, heterochronic | GO:0040034 | 2.2E-03 | 2.652 |
| Others |  |  |  | 173.5 |
